# Single cell transcriptomics reveals cell type specific features of developmentally regulated responses to lipopolysaccharide between birth and 5 years

**DOI:** 10.3389/fimmu.2023.1275937

**Published:** 2023-10-17

**Authors:** James F. Read, Michael Serralha, Jesse D. Armitage, Muhammad Munir Iqbal, Mark N. Cruickshank, Alka Saxena, Deborah H. Strickland, Jason Waithman, Patrick G. Holt, Anthony Bosco

**Affiliations:** ^1^ Asthma and Airway Disease Research Center, University of Arizona, Tucson, AZ, United States; ^2^ Telethon Kids Institute, The University of Western Australia, Perth, WA, Australia; ^3^ School of Biomedical Sciences, The University of Western Australia, Nedlands, Western Australia, Australia; ^4^ Genomics WA, Joint Initiative of Telethon Kids Institute, Harry Perkins Institute of Medical Research and The University of Western Australia, Nedlands, WA, Australia; ^5^ UWA Centre for Child Health Research, The University of Western Australia, Nedlands, WA, Australia; ^6^ Department of Immunobiology, The University of Arizona College of Medicine, Tucson, AZ, United States

**Keywords:** single cell genomics, scRNA-Seq, toll-like receptors, lipopolysaccharide, poly(I:C), interferon, proinflammatory, cord blood

## Abstract

**Background:**

Human perinatal life is characterized by a period of extraordinary change during which newborns encounter abundant environmental stimuli and exposure to potential pathogens. To meet such challenges, the neonatal immune system is equipped with unique functional characteristics that adapt to changing conditions as development progresses across the early years of life, but the molecular characteristics of such adaptations remain poorly understood. The application of single cell genomics to birth cohorts provides an opportunity to investigate changes in gene expression programs elicited downstream of innate immune activation across early life at unprecedented resolution.

**Methods:**

In this study, we performed single cell RNA-sequencing of mononuclear cells collected from matched birth cord blood and 5-year peripheral blood samples following stimulation (18hrs) with two well-characterized innate stimuli; lipopolysaccharide (LPS) and Polyinosinic:polycytidylic acid (Poly(I:C)).

**Results:**

We found that the transcriptional response to LPS was constrained at birth and predominantly partitioned into classical proinflammatory gene upregulation primarily by monocytes and Interferon (IFN)-signaling gene upregulation by lymphocytes. Moreover, these responses featured substantial cell-to-cell communication which appeared markedly strengthened between birth and 5 years. In contrast, stimulation with Poly(I:C) induced a robust IFN-signalling response across all cell types identified at birth and 5 years. Analysis of gene regulatory networks revealed IRF1 and STAT1 were key drivers of the LPS-induced IFN-signaling response in lymphocytes with a potential developmental role for IRF7 regulation.

**Conclusion:**

Additionally, we observed distinct activation trajectory endpoints for monocytes derived from LPS-treated cord and 5-year blood, which was not apparent among Poly(I:C)-induced monocytes. Taken together, our findings provide new insight into the gene regulatory landscape of immune cell function between birth and 5 years and point to regulatory mechanisms relevant to future investigation of infection susceptibility in early life.

## Introduction

Newborns experience remarkable environmental change as they transition from a protected, tolerogenic intrauterine environment to the outside world with an abundance of stimuli and pathogens (1). The neonatal immune system has evolved unique functional characteristics suited to the challenges of perinatal life (2). For example, neonatal myeloid cell cytokine production is skewed to promote Th_2_ and Th_17_ responses, while those that promote Th_1_ and Type I interferon (IFN) responses are attenuated (3–7). Within the lymphoid compartment, neonatal T cells arise from distinct hematopoietic stem cell populations (8), express more broadly reactive T cell receptors (9, 10), display distinct epigenetic patterns (11, 12), and exhibit impaired memory capacity (13, 14) compared to adult counterparts (2). Importantly, cord blood-derived T cells demonstrate enhanced responses to innate immune signals (2), including those associated with activation of the Toll-like Receptor (TLR) family (15, 16). Despite these functional adaptions, newborns are nonetheless highly susceptible to developing severe disease following microbial infections.

Innate immune responses are triggered by the binding of evolutionarily conserved pathogen-associate molecular patterns (PAMPs) to germline encoded pathogen recognition receptors (PRRs) of the innate immune system. The Toll-Like Receptor (TLR) family are the most well characterized PRRs, and are expressed on the cell surface (e.g., TLRs 1, 2, 4, 5, 6) or in intracellular endosomes (e.g., TLRs 3, 7, 8) (17). Cell surface TLRs bind to bacterial cell membrane/wall components and viral proteins, whereas the intracellular TLRs bind to nucleic acids (17). Downstream signalling of TLR activation is predominantly mediated by either Myeloid differentiation factor 88 (MyD88) and/or Toll-Interleukin 1 Receptor-domain-containing adapter-inducing IFN-β (TRIF) (17, 18). TLR-signaling induces subsequent effector gene expression programs following engagement of specific transcription factor (TF), including Nuclear Factor kappa-B (NF-κB) and members of the IFN Regulatory Factor family (e.g. IRF3/7) (17, 18), resulting in the promotion of proinflammatory (e.g. IL-1β, IL-6) and Type I IFN/antiviral (IFNα/β) response programs, respectively (17–20). TLR4 mediates immune responses following detection of lipopolysaccharide (LPS), a cell wall component of Gram-negative bacteria. Uniquely among TLRs, TLR4 ligation triggers both the MyD88-dependent proinflammatory and TRIF-dependent Type I IFN response pathways (17). TLR3 recognises viral RNA and triggers TRIF-dependent Type I IFN production (17, 18).

Innate immunity has traditionally been viewed as a first line of defence capable of responding rapidly and non-specifically to pathogen encounters that lacks memory and therefore cannot mediate resistance to reinfection. This view has been challenged by the demonstration that exposure to vaccines, infections, or microbial products can induce prolonged epigenetic and functional changes in innate immune cells that provide enhanced, non-specific protection to subsequent encounters with the same pathogen or an unrelated pathogen (21). This evolving view of early life immunity provides motivation for more studies to further our understanding of how innate immune function at birth is programmed during the first years of life, a crucial period of heightened plasticity and window of susceptibility for the development of chronic diseases. The advent of single cell genomics enables a deeper understanding of the cell type specific, stimuli specific, and age-related changes that underlie the development and regulation of innate immune function in early life. Here, we deploy these powerful tools to analyse matched cord/peripheral blood mononuclear cell (C/PBMC) samples collected at birth and five years of age from two donors to provide a unique level of insight into the developmental regulation of innate immune function at birth versus early childhood at single cell resolution.

## Materials and methods

### Study subjects

The study was designed to assess matched birth (CBMC) and 5 years (PBMC) blood samples following LPS and Polyinosinic:polycytidylic acid (Poly(I:C)) treatment, along with matched untreated controls, from two donors (one male, one female). The samples were curated from the Childhood Asthma Study (CAS) cohort, a prospective birth cohort described previously (22). Cord blood samples were collected from healthy, full-term, singleton births. Matched 5-year samples were collected from the same donor by home visit close to their 5^th^ birthday. Ethics was approved by The University of Western Australia (reference RA/4/1/7560), and fully informed parental consent was obtained for each subject.

### *In vitro* cell culture and innate immune stimulation

Cryopreserved CBMC/5yr PBMC samples were thawed and stimulated with 1ng/ml LPS (Enzo Biochem) or with 50μg/ml Poly(I:C) (InvivoGen), alongside an untreated control, and culture plates were incubated at 37°C (5% CO_2_) for 18 hours (Extended Methods). LPS is a bacterial cell wall component and a quintessential TLR4 ligand (23). Poly(I:C) is a synthetic analogue of double-stranded RNA (dsRNA) and a potent activator of TLR3 and other viral nucleic acid sensing receptors (24). Each cryopreserved sample was cultured separately so that matched stimuli/control samples were processed alongside each other in a batch. Following cell culture, samples were re-suspended at target concentration of 2000 cells/μl. Post-culture viability is recorded in **Table S1**.

### Library preparation and sequencing

Single cells were processed on Chromium using the Chromium Next GEM Single Cell 3’ Kit v3.1 (4 reactions, PN-1000269, 10X Genomics) on Chip G (PN-1000127, 10x Genomics) according to the manufacturer’s protocol with targeted recovery of 5,000 cells per channel. Libraries were sequenced on the NovaSeq 6000 platform in a single batch.

### Alignment and initial quality control

Raw fastq.qz files were processed with the CellRanger Toolkit (Version 6.1.1, 10x Genomics) and the Human GRCh38 genome assembly (refdata-gex-GRCh38-2020-A) was used as the reference genome. The CellRanger count pipeline was run with standard parameters. CellRanger web_summary outputs were assessed with no alerts (warnings or errors) recorded for any sample. Selected CellRanger outputs are recorded in **Table S1**; briefly, this project generated (on average) 5527.17 cells per sample with 63,098.75 mean reads per cell and a median of 1980.17 genes detected per cell, as estimated by CellRanger. The raw count matrix, and corresponding barcodes and features, were used for downstream QC and analysis, and these are available via the Gene Expression Omnibus (GSE232186).

### Sample pre-processing and quality control

Count matrices were imported into the R statistical environment (version 3.6.2) for quality control (QC) and analysis. The open-source R toolkit Seurat (version 3.2.0) (25) was primarily used for pre-processing and data exploration (Extended Methods). Briefly, cells/UMIs were excluded if they had low feature counts (approximately < 2000) according to dynamic thresholding of the count distribution or if their mitochondrial gene content was greater than three median absolute deviations (MADs) above the median. Features were filtered to only those expressed in at least 0.01% of cells. Doublets were detected and removed with DoubletFinder (26) and the cell cycle phase was estimated with the CellCycleScoring function (Seurat) using known cell cycle related genes. Pre-processing and quality control metrics are recorded in **Table S1**.

### Integration, annotation, and dimensionality reduction

Seurat (25) was used to integrate individual pre-processed samples (Extended Methods). Briefly, 2,000 anchors were identified with the FindIntegrationAnchors function with k.anchor = 5, reduction = “rpca”, and dims = 1:30. These anchors were input into the IntegrateData function to integrate the data. Individual cells were annotated with Azimuth (27) which provides reference-based mapping to unbiasedly annotate scRNA-Seq profiles. The human PBMC reference data set was used as the reference; a CITE-seq dataset with gene expression for hundreds of thousands of cells alongside a large panel of antibodies to accurately identified cell types present in the PBMC (27). The level 2 cell type resolution was used, and cells were excluded if they had a score less than 0.5. Uniform Manifold Approximation and Projection (UMAP) was employed for dimensionality reduction. First, the integrated data set was scaled and centered with the ScaleData function, and the first 30 principal components were calculated with the RunPCA function. Although non-linear dimensionality reduction methods such as UMAP are widely used to visualize relationships between cells within high dimensional data in 2-Dimensional space (e.g., cell type clusters), these methods often do so at the cost of preservation of the local and/or global structure of the data (28). We tested whether the plots generated from our UMAP analysis were robust to different values of two of the most influential parameters, the number of nearest neighbors (n.neighbors) and the minimum distance (min.dist) between cells. Plots were created with combinations of nearest neighbour values of 5, 10, 15, 20, 30, 40, 50 and minimum distance values of 0.1, 0.2, 0.3, 0.4, and 0.5. These values were chosen as they span the suggested values for these parameters. The UMAP plot with nearest neighbour value 20 and a minimum distance values of 0.3 was selected as a representative to display integrated cell type clustering with Azimuth annotations, group variables, and marker gene expression intensities.

### Differential gene expression and pathways analysis

To identify differentially expressed genes (DEGs) between LPS/Poly(I:C) and corresponding unstimulated controls for each cell type, we employed MAST (29) via the FindMarkers function from Seurat, and included cellular detection rate, mitochondrial gene proportion, and cell cycle phase as covariates. As each donor represents a different biological sex (male, female), this variable was also included as a latent variable. This approach was selected to accommodate our small sample size (two biological donors) (See Extended Methods). Genes were considered differentially expressed if they recorded a Bonferroni-corrected *p* value less than 0.01 and an average Log_2_ fold-change in expression greater than 0.25 (upregulated) or less than -0.25 (downregulated). To assess whether a discrepancy between the number of CD14^+^ monocytes from each donor impacted the differential expression analysis, we randomly selected an even number of CD14^+^ monocytes (1,000 per donor) from each donor and applied MAST analysis as above. Significantly enriched pathways associated with DEGs between cell type/stimuli groups were identified with enrichR (30) in R to query biologically relevant annotated gene sets from the Reactome (31), KEGG (32), and Gene Ontology (33) databases.

### Pseudotime trajectory inference

We applied Monocle3 (34) to infer stimuli-related activation trajectories from transitional cellular states present in the data (Extended Methods). For each analysis, only the raw counts from cells relevant to that comparison (e.g., CBMC/5yr PBMC untreated and LPS-treated monocytes) were included. Regions enriched with unstimulated controls were selected as pseudotime start points so that trajectories extended into stimuli-activated regions.

### Gene Regulatory Network (GRN) analysis and *in silico* perturbations

We employed CellOracle (35) (version 0.12.0) to build GRNs and identify key TF drivers and their corresponding target genes for selected cell type/stimuli groupings. For this analysis, SCANPY (36) (1.9.3) was used for pre-processing and force directed graph construction and CellOracle was run with Python (3.10.6) on Ubuntu 22.04.1 (Extended Methods). The dataset was filtered to include the top 3000 most variable genes for each comparison, and the data was normalized, log transformed and scaled (Extended Methods). Separate analyses were run from raw counts for each cell type/stimuli comparison and group specific GRNs were constructed from the base Human promoter GRN provided, and the Monocle3-defined pseudotime values for each cell were included for analysis. GRNs were used to perform *in silico* TF perturbations of IRF1, IRF7, and STAT1 to simulate the changes in cellular states after nullifying the regulatory effects of these TFs.

### Ligand-receptor interaction analysis

We employed CellCall (37) to identify putative ligand-receptor (L-R) communication between selected cell types following LPS- and Poly(I:C)-induced activation. For each stimuli/age comparison, the genes were filtered to the top 3000 most variable genes (compared to untreated control) and single cell profiles were restricted to selected cell types of interest. Raw counts were use as input and the transcriptional communication profile was constructed with the TransCommuProfile function. The LR2TF function was used to extend the analysis by capturing putatively activated TFs downstream of receiver cell receptor binding of sender cell ligands for reciprocal communication between CD14^+^ monocytes and naïve CD4^+^ T cells.

## Results

### Cord and 5yr blood-derived single cell transcriptomic profiles display age-related compositional differences

We cultured matching CBMC and 5yr PBMC samples from two donors in the presence or absence of LPS or Poly(I:C) and generated single cell transcriptomic profiles (n=12) to investigate the immune responses elicited. Following pre-processing and quality control, 57,908 single cells were included for downstream analysis (mean = 4826.7 cells per sample) with an average of 7400.4 counts and 2190.3 genes detected per cell (**Table S1**).

**Figure 1:**
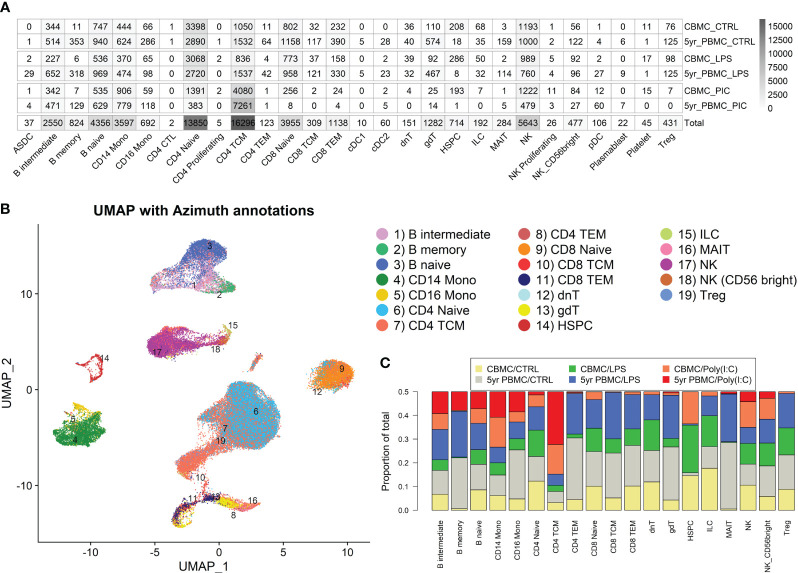
Overview of single cell RNA-Seq profiles generated. **(A)** Heatmap showing the number of cells identified for each Azimuth-annotated cell type, stratified by stimuli/age group. The final row of indicates the total number of cell detected for that cell type. **(B)** UMAP plot generated from the integrated data, overlaid with Azimuth annotations. **(C)** Stacked bar plot demonstrating the proportional contribution of cell numbers among stimuli/age groups.

CD4^+^ naive and central memory T cells, NK cells, naïve B cells, CD8^+^ naïve T cells, and CD14^+^ monocytes had the greatest cell numbers of the 28 cell types detected with Azimuth reference-based annotation mapping (27) (**Figure 1A**). The contribution of cell numbers was generally comparable between donors, although some variation was present among some cell types (e.g., Donor 1 had 1,127 CD14^+^ monocytes whereas Donor 2 had 2,470) (**Figures S1A–C**). Negligible cell numbers were detected for several cell types (e.g., dendritic cells subsets, plasmablasts, and platelets) (**Figure 1A**), and these were not considered in downstream analysis. We performed UMAP analysis of the integrated data set to visualize the single cell gene expression profiles in lower dimensional space. This analysis demonstrated clustering according to the major immune subsets, displaying prominent populations of CD4^+^ and CD8^+^ T cells, B cells, NK cells, and monocytes, alongside a smaller population of hematopoietic stem and progenitor cells (HSPC) (**Figure 1B**). As, expected, HSPCs were more prominent among CBMC samples and memory B cells were largely restricted to 5yr PBMC samples (**Figures 1A, C**, **S1C**). Several T cell subtypes, including γδT cells and MAIT cells, also had greater cell counts at 5 years compared to birth, although they were rare or non-existent among Poly(I:C) treated samples (**Figurea 1A, C**, **S1C**). Analysis of the estimated cell cycle phase indicated that the majority of monocytes were in G1 phase and the majority of HSPCs were in S phase (**Figure S1C**). Plotting the expression intensity of canonical cell type marker genes over the UMAP coordinates confirmed Azimuth annotation of the major mononuclear cell subtypes (**Figure S1D**). To assess whether our UMAP analysis was robust to a range of parameters, we generated UMAP plots with several combinations of two of the most influential parameters, the number of nearest neighbors and the minimum distance between cells, and the clustering was generally comparable between plots (**Figure S2**).

### Differential expression analysis reveals an LPS-specific division of labor among myeloid and lymphoid immune cell compartments

**Figure 2:**
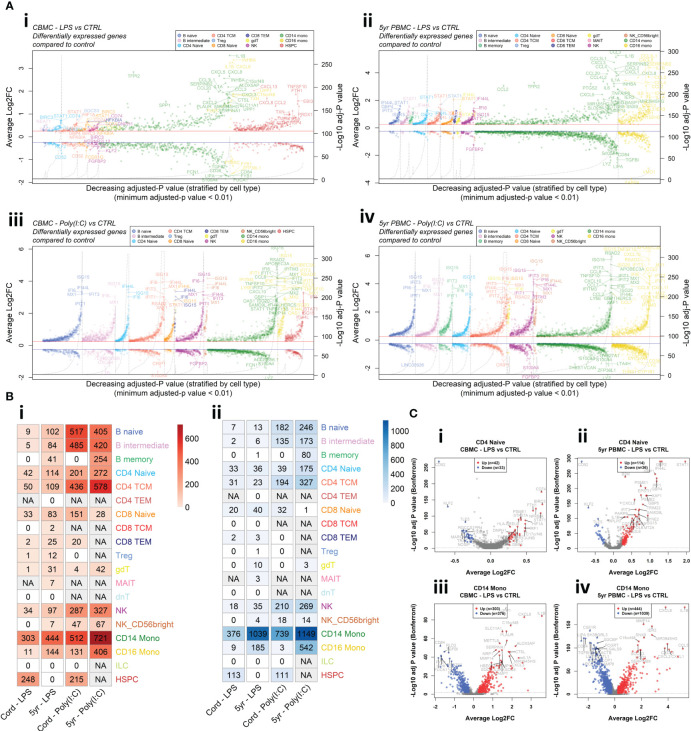
Differential expression analysis between stimulated samples and matched unstimulated controls. **(A)** Aligned volcano plots displaying differentially expressed genes compared to unstimulated controls for the LPS-stimulated CBMC (i), LPS-stimulated 5-year PBMC (ii), Poly(I:C)-stimulated CBMC (iii), and Poly(I:C)-stimulated 5-year PBMC (iv) comparisons. Each point represents a differentially expressed gene (Adjusted-*p* value < 0.01 (Bonferroni correction), average Log_2_FC > 0.25) ordered by decreasing Bonferroni corrected *p* value and stratified by cell type (x-axis). The left y-axis corresponds to points on the plots and shows the average Log_2_ fold change for that gene/cell type. The right y-axis corresponds to dashed black line and represents the -Log_10_ Bonferroni corrected *p* value. Solid red line (blue line) represents an average Log_2_ fold change of 0.25 (-0.25). **(B)** Heatmap showing the number of significant differentially expressed genes that were upregulated (i) and downregulated (ii) for each group compared to their corresponding unstimulated control. Columns correspond to the age/stimuli group, where cord refers to CBMC samples and 5yr refers to 5yr PBMC samples. Increased color intensity corresponds to a greater number of differentially expressed genes for that comparison and comparison with insufficient cell numbers for analysis a denoted as not applicable (grey). **(C)** Volcano plots of the comparison of LPS versus unstimulated control of naïve CD4^+^ T cells from CBMC (i) and 5yr PBMC (ii) samples, and CD14^+^ monocytes from CBMC (iii) and 5yr PBMC (iv) samples. The x-axis shows the average log_2_ fold change and the y-axis shows the -Log_10_ Bonferroni-corrected *p* value. The dashed grey line indicates a Bonferroni-corrected *p* value of 0.01. Points colored red and blue represent gene with are considered significantly upregulated and downregulated, respectively.

To investigate changes in gene expression following *in vitro* exposure to LPS and Poly(I:C) we employed MAST (29) analysis to identify DEGs (Log_2_FC > |0.25| and Bonferroni-corrected *p* < 0.01) between stimulated cells and corresponding unstimulated controls for each cell type. In general, changes in gene expression magnitude induced by Poly(I:C) were greater than LPS for both CBMC and 5yr PBMC samples (**Figures 2A, B**, **S3A, B**). Genes encoding proinflammatory cytokines, such as *IL1B* and *CXCL8*, were prominently upregulated by monocytes following LPS stimulation of CBMCs, and this proinflammatory gene expression signature strengthen at 5 years of age (**Figures 2A–C**, **S3A**). Additionally, there was a marked increase in the number of DEGs by CD16^+^ monocytes between CBMC and 5yr PBMC samples (**Figures 2A, B**, **S3A**). Interestingly, HSPCs demonstrated a substantial gene expression response to stimuli at birth with a distinctive LPS-induced transcriptional profile – which includes *CXCL8* and *CXCL13* – compared to the IFN-related Poly(I:C)-stimulated HSPC profile (**Figures 2A, B**, **S3B**). Strikingly, several CBMC-derived T and B cell subsets exhibited upregulation of IFN-related genes, such as *IRF1* and *STAT1*, following LPS stimulation at birth (**Figures 2A, C**, **S3A**). Furthermore, we found that IFN-related genes exhibited enhanced upregulation in lymphocytes isolated from LPS-treated 5yr PBMCs compared to LPS-treated CBMCs (**Figures 2A–C**, **S3A**). Individual volcano plots of the LPS-induced gene expression comparisons for naïve CD4^+^ T cells and CD14^+^ monocytes are shown in **Figure 2C** to highlight the elevated upregulation of IFN-related genes by CD4^+^ T cells at 5yrs compared to birth and the substantial proinflammatory monocyte response (**Figure 2C**). In contrast to the LPS-stimulated samples, Poly(I:C) induced a strong IFN-mediated response in all cell types in the CBMC and 5yr PBMC samples, exemplified by upregulation of IFN Stimulated Gene 15 (*ISG15*) (**Figures 2A, B**, **S3A**). Assessment of the overlap of upregulated genes showed approximately twice as many genes were conserved between CBMC and 5yr PBMC responses to Poly(I:C) compared to LPS (**Figure S3C**). Differential gene expression results for all cell types and comparisons have been compiled together and are presented in the **Supplementary Data**. As mentioned above, Donor 2 contributed substantially more CD14^+^ monocytes to the data set than Donor 1. To assess whether this discrepancy impacted the differential expression analysis, we randomly selected 1,000 CD14^+^ monocytes from each donor and re-run the analysis. The results show a substantial overlap (~90%) between the top dysregulated genes (ordered by adjusted-*p* value) identified for the analysis of all CD14^+^ monocytes and the randomly selected (n=1,000/donor) subset (**Figure S4**).

Overall, the differential expression analysis demonstrates that LPS treatment of mononuclear cells induces contemporaneous upregulation of classical proinflammatory genes by monocytes and IFN-related genes by lymphocytes. Furthermore, these partitioned responses show apparent strengthening between samples collected at birth and 5 years of age. Importantly, matched samples treated with Poly(I:C) demonstrated uniform upregulation of IFN-related genes across all cell types with a comparable number and change in expression magnitudes of dysregulated genes between birth and 5 years.

### IFN-signaling pathways are activated by LPS in lymphocytes at birth and intensify by 5 years

**Figure 3:**
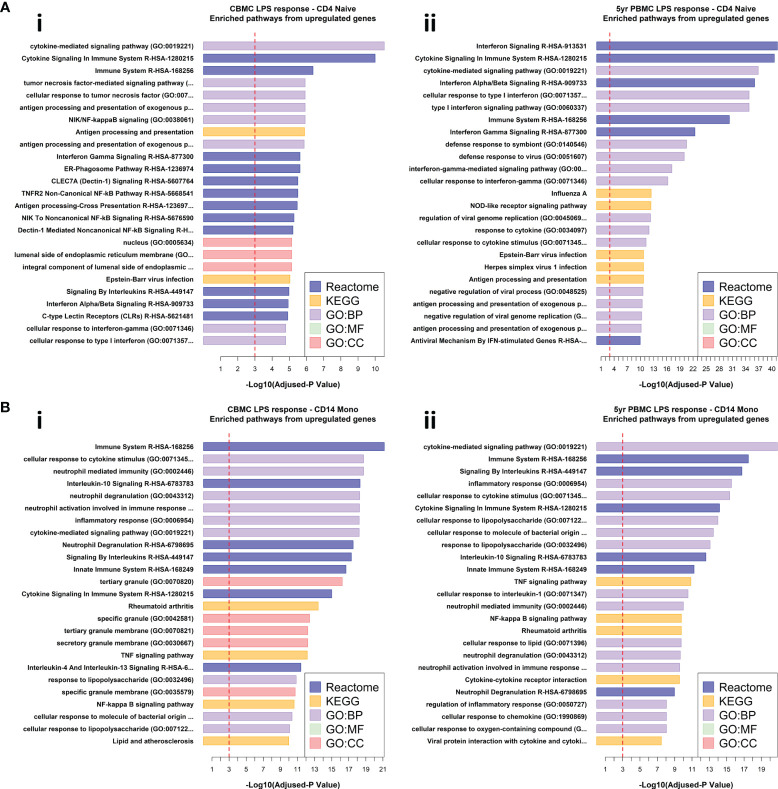
Pathway analysis of differentially expressed genes. **(A)** Horizontal bar plots of significantly enriched pathways from upregulated genes found for the comparison of CD4^+^ naïve T cells stimulated with LPS versus corresponding unstimulated control from CBMC (i) and 5yr PBMC (ii) samples. **(B)** Horizontal bar plots of significantly enriched pathways from upregulated genes found for the comparison of CD14^+^ Monocytes stimulated with LPS versus corresponding unstimulated control from CBMC (i) and 5yr PBMC (ii) samples. For the above plots, the x-axis shows the -Log_10_ adjusted-*p* value associated with pathways enrichment, the dashed red line indicates an adjusted-*p* value of 0.001. Results are ordered from top by decreasing adjusted-*p* value for significantly enriched pathways identified from the Reactome, KEGG, and Gene Ontology (GO) databases. BP, Biological Process; MF, Molecular Function; CC, Cellular Compartment.

We leveraged the Reactome (31), KEGG (32), and Gene Ontology (33) databases to determine significantly enriched biological pathways from our DEG lists. LPS-induced upregulated genes from CBMC-derived naïve CD4^+^ T cells exhibited enrichment of cytokine signaling pathways, including IFN-signaling, among several immune-related pathways (**Figure 3A**). At 5 years, IFN-related pathways were among the dominant pathways identified from upregulated genes of LPS-induced naïve CD4^+^ T cells (**Figure 3A**), aligning with the findings from our differential expression analysis. Comparable results were observed for naïve B cells, naïve CD8 T cells, and NK cells (**Figures S5A, B**). Upregulated genes from LPS-induced CD14^+^ monocytes exhibited similar pathways enrichment from CBMC and 5yr PBMC samples, including several pathways associated with antibacterial responses and specific LPS response pathways (**Figure 3B**). IFN-related pathways dominated the significantly enriched results from the upregulated gene lists for all cell types detected from Poly(I:C)-treated CBMC and 5yr PBMC samples (**Figures S5C–E**).

Taken together, analysis of DEGs and pathways enrichment suggests that the immune response to Poly(I:C), and thus some viral pathogens, is to some degree hardcoded at birth to elicit a substantial IFN-mediated response from all immune cell types, and this biological feature remains relatively unchanged at 5 years of age. In contrast, LPS-induced CBMC/PBMCs demonstrate a division of labour whereby cells of the myeloid immune compartment express proinflammatory genes commonly associated with antibacterial responses at birth and age 5 whilst lymphocytes upregulate detectable quantities of IFN-signaling related genes/pathways at birth, which demonstrate a subsequent and substantial increase in prominence, and presumably function, at 5 years of age.

Following our primary analysis of gene expression above, we focussed our subsequent secondary analysis of inferred activation trajectories, gene regulatory networks, and ligand-receptor interactions on CD14^+^ monocytes and naïve CD4^+^ T cells as exemplars of the myeloid and lymphoid immune compartment, respectively.

### Pseudotime mapping of cell differentiation trajectories demonstrates cell type- and age-specific immune activation

The various intermediary cellular states captured by single cell transcriptomics can be used to infer pseudotime trajectories and reconstruct biological processes (38). For this purpose, we employed monocle3 (34) to infer dynamic trajectories underlying LPS and Poly(I:C) activation of CD14^+^ monocytes and naïve CD4^+^ T cells in CBMC and 5yr PBMC samples. Our analysis shows that CD14^+^ monocytes from unstimulated CBMC and 5yr PBMC control samples cluster together, indicating that baseline expression is relatively similar at birth and in early childhood (**Figure S6A**). However, stimulation with LPS promotes distinct activation endpoints with respect to the age at which the sample was collected (**Figure S6A**). In contrast, treatment with Poly(I:C) produces a single activation trajectory for CD14^+^ Monocytes isolated from CBMC and 5yr PBMC samples (**Figure S6B**). The unstimulated controls from CBMC- and 5yr PBMC-derived naïve CD4^+^ T cells cluster separately suggesting distinct baseline profiles, and subsequently produced distinct activation states following LPS treatment (**Figure S6C**). Similar trajectory characteristics were observed for CD8^+^ T, B, and NK cells stimulated with LPS (**Figures S6D–F**), although there was minimal distinction between LPS-treated and control B and NK cells from CBMC samples – likely explained by their relatively small gene expression change following LPS-activation. Gene expression profiles from T, B, and NK cells treated with Poly(I:C) exhibited a stimuli effect substantially greater than the difference between the CBMC and 5yr PBMC responses, resulting in the inability to fit a biologically meaningful trajectory to the data (**Figure S6F**). Summarizing the above analysis, these results indicate that LPS-treated CD14^+^ monocytes may exhibit distinct age-related (CBMC/5yr PBMC) activation branches from common baseline profiles, whereas CD4^+^ naïve T cells exhibit distinct age-related baseline profiles and subsequent activation trajectories.

### Gene regulatory network analysis identifies IRF1 and STAT1 as key regulators of LPS-induced lymphocyte response

**Figure 4:**
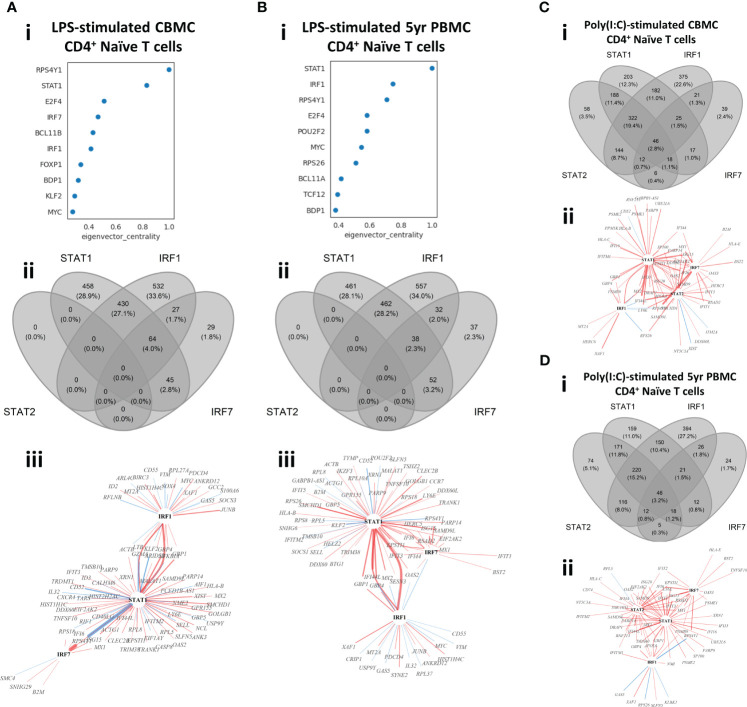
Identification of master regulators of *in vitro* stimulation responses of CBMC- and 5yr PBMC-derived naïve CD4^+^ T cells. **(A, B)** Output plots from CellOracle (34) network analysis showing transcription factors and target genes ranked by eigenvector centrality for LPS-induced naïve CD4^+^ T cells (i) isolated from CBMC **(A)** and 5yr PBMC **(B)** samples (panels continue downward). Venn diagrams (ii) depict the overlap in target genes between the selected IFN-related transcription factors identified in the CellOracle analysis for the corresponding analysis above; all target genes which were significantly associated (*p* value < 0.01) with one of the IFN-related TFs were included in the analysis. TF-TG wiring diagrams (iii) illustrate the interrelationship between the selected IFN-related TF and the top 100 connections to target genes by connection strength (absolute coefficient) for the corresponding analysis above. The regulators (TFs) are identified by black text and target genes are identified by grey text. Red connections indicate positive relationships (increased TG activity) and blue connections indicate a negative relationships (suppressed TG activity). The width of the connecting line illustrates relative strength with the network shown. **(C, D)** Venn diagrams (i) and TF-TG wiring diagrams (ii) for the analysis of Poly(I:C)-induced naïve CD4^+^ T cells isolated from CBMC **(C)** and 5yr PBMC **(D)** samples. Analysis and plotting parameters are identical to those used for the LPS-induced response shown in **(A)** and **(B)**.

Genes are expressed by the coordinated action of cis-regulatory elements and TFs. Construction of context specific Gene Regulatory Networks (GRN) allows investigation of the relationship between TFs and their target genes (TGs). To this end, we constructed context-specific GRNs with CellOracle (35) to identify predicted TFs which act as putative master regulators of the innate immune responses investigated in this study. This approach identified the key IFN-signaling drivers STAT1, IRF1, and IRF7 among the top TFs identified from LPS-induced naïve CD4^+^ T cell isolated from CBMC samples (**Figure 4A**). Furthermore, STAT1 and IRF1 were the top regulators of the 5yr naïve CD4^+^ T cell response to LPS, using eigenvector centrality as the metric. Indeed, these TFs were among the top regulators of all LPS-induced lymphocytes assessed in this study from CBMC and 5yr PBMC samples, and this result was consistent across metrics used to assess the GRN (eigenvector, betweenness, or degree centrality) (**Figures S7A–D**). Several known mediators of myeloid inflammation (e.g., FOS, JUN, ATF3) were identified as the key regulators of the LPS-induced monocyte responses (**Figure S7E**).

As we previously identified IFN-related genes and pathways characterized the lymphocyte response to LPS, we further investigated the role of IRF1, IRF7, and STAT1, alongside related TF STAT2, within our experimental context. We used Venn diagrams to visualize the overlap of all target genes with significant connections (*p* value < 0.01) to these TFs (**Figures 4A, B**). This analysis demonstrated similar overlap in target genes between CBMC- and 5yr PBMC-derived naïve CD4^+^ T cells stimulated with LPS. Additionally, IRF1 and STAT1 accounted for approximately 90% of TG connections and the response network was independent of STAT2 activity (**Figures 4A, B**). We next plotted wiring diagrams of the top 100 TG connections for the selected TFs to assess the relationships among the most strongly regulated interactions (**Figurez 4A, B**). IRF1 and STAT1 were strongly connected to each other, reflecting the functional association of IRF1 with STAT1 homodimers (39), and STAT1 demonstrated the greatest number of connections for LPS-induced naïve CD4^+^ T cells birth and 5 years (**Figures 4A, B**). Interestingly, IRF7, which was only peripherally connected at birth, integrates more strongly with STAT1 and IRF1 via IFN-related TGs (e,g., *ISG15*, *IFIT3*, *IFI6*) at 5 years (**Figures 4A, B**). IRF1, IRF7, STAT1, and STAT2 were consistently among the top drivers of the Poly(I:C)-induced lymphocyte response following identical analysis (**Figures S8A–D**). The overlap of significantly connected TG was similar between Poly(I:C)-induced naïve CD4^+^ T cells from CBMC and 5yr PBMC samples and the contribution of STAT2 stands in stark contrast to its absence in the corresponding LPS response analysis (**Figures 4C, D**). Additionally, wiring diagrams of the most connected TG were dominated by the interaction between IRF7, STAT1, and STAT2 and IFN-associated target genes, and this was comparable between birth and 5 years of age (**Figures 4C, D**).

A core functionality of CellOracle is the ability to perform *in silico* perturbations to simulate TF knock-out and assess the outcome on cellular states (35). Simulated knock-out of *IRF1* or *STAT1* results in a reversal of the activation trajectory of LPS-induced naïve CD4^+^ T cells from CBMC and, to a lesser extent, 5yr PBMC samples, whereas knock-out of IRF7 primarily affects 5yr PBMC-derived samples (**Figure S9**), aligning with inferences from the corresponding wiring diagrams (**Figures 4A, B**). These findings demonstrate that IRF1 and STAT1 are central drivers of the lymphocyte response to LPS in early life and suggest an important developmental role for IRF7.

### Ligand-receptor interaction analysis reveal dynamic immune cell crosstalk patterns at birth and 5 years

**Figure 5:**
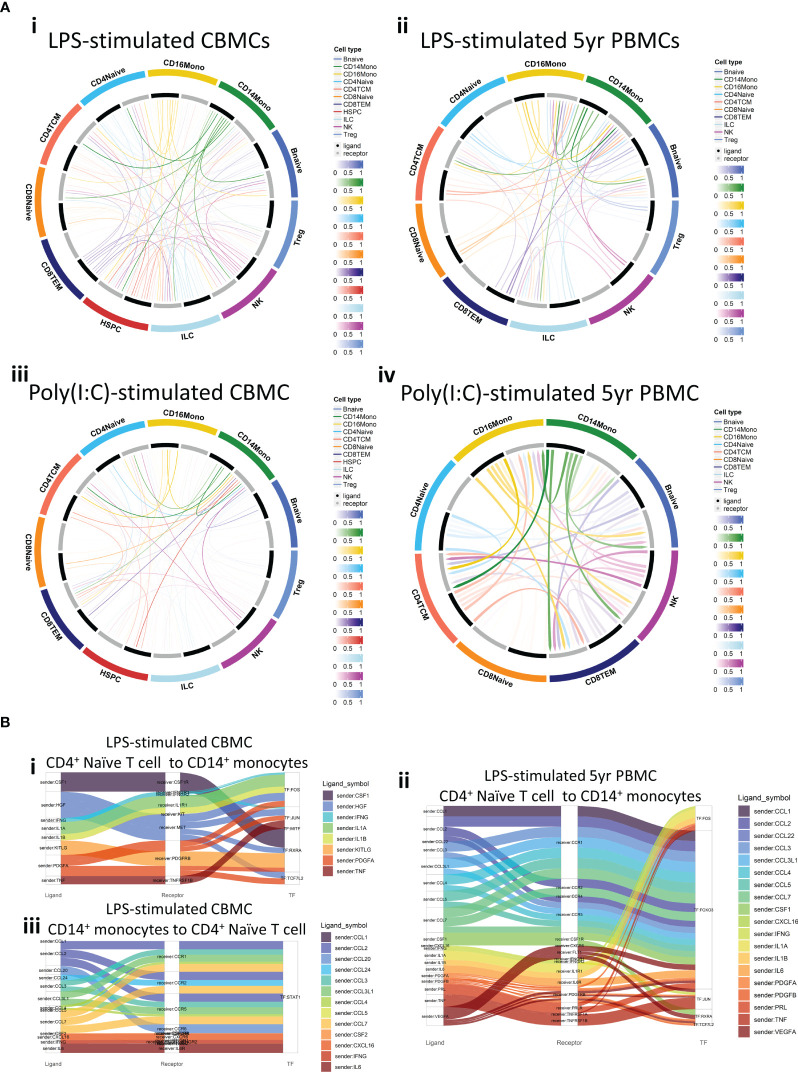
Cell-to-cell communication and subsequent transcription factor activation. **(A)** Circos plots of ligand-receptor communication for selected cell types for LPS-stimulated CBMC (i) and 5yr PBMC (ii) samples and Poly(I:C)-stimulated CBMC (iii) and 5yr PBMC (iv) samples. Cell type ligands are represented by black bars, cell type receptors are represented by grey bars, and the strength of the signal is represented by the color intensity. **(B)** Sankey plots demonstrating the cell-to-cell communication from ‘sender’ cell ligands (left column) to ‘receiver’ cell receptors (central column) and the subsequent transcription factor putatively activated from the ligand-receptor interaction (right column). Plots shows the communication from naïve CD4^+^ T cells to CD14^+^ monocytes for LPS-induced CBMC (i) and 5yr PBMC (ii) samples, and the reverse communication (CD14^+^ monocytes to naïve CD4^+^ T cells) for LPS-induced CBMC samples (iii). TF, Transcription factor.

PAMP recognition by PRRs provokes downstream production of effector ligands by activated immune cells. These ligands enact subsequent intercellular communication by binding to their cognate receptors on other immune cells, prompting a signal cascade which mediates downstream transcription factor activity. We employed CellCall (37) to predict cell-cell communication pairings and capture putative signaling cascades following LPS- or Poly(I:C)-induced activation of selected cell types in this study. This approach revealed complex interconnectedness within lymphoid subsets and between lymphoid subsets and monocytes from stimulated samples (**Figure 5A**). HSPCs also demonstrated ligand-receptor pairing (**Figure 5A**), suggesting they participate in the CBMC response to stimuli investigated in this study. Interestingly, receptors of several 5yr PBMC-derived lymphocyte subsets (e.g., naïve CD4^+^ and CD8^+^ T cells) recorded limited or absent incoming signals, which were active in corresponding CBMC-derived samples (**Figure 5A**).

We next focussed on the receptor-ligand interactions between LPS-induced naïve CD4^+^ T cells and CD14^+^ monocytes. At birth, CD4^+^ T cell-associated cytokine and growth factor ligands (e.g., IFNG, IL1A/B, PDGFA) were identified as inducers of several TFs, including the activating protein-1 (AP-1) TF complex members FOS and JUN [**Figure 5B**(i)]. JUN and FOS are also predicted to be activated by naïve CD4^+^ T cell ligands in CD14^+^ monocytes at 5 years, and there is prominent additional signaling from suite of chemokine (e.g., CCL1-5) promoting FOXO3 activity [(**Figure 5B**(ii)]. Analysis of the reverse direction identified interactions between CD14^+^ monocytes ligands, including CCL chemokines, IFNG, and IL6, and naïve CD4^+^ T cell receptors, all of which putatively induced STAT1 activity which may in part explain the STAT1 prominence within the LPS-induced naïve CD4^+^ T cell regulatory networks observed in **Figure 4**. No interactions from CD14^+^ monocyte ligands to naïve CD4^+^ T cell receptors were detected for 5-year PBMC samples from this analysis. Taken together, these intracellular signaling analyses reveal complex ligand-receptor interactions between immune cell types isolated from CBMC and 5yr PBMC samples treated with LPS and Poly(I:C), and that the relationship of these interactions are subject to change between birth and 5 years of age.

## Discussion

The neonatal immune system exhibits unique functional characteristics that are tailored to the challenges of perinatal life (40). Here, we employed single cell RNA-Seq to deeply profile innate immune responses to Poly(I:C) and LPS in longitudinally matched CBMC/PBMC samples collected from two donors at birth versus age five years. We found that Poly(I:C) induced a robust response across all cell types regardless of age. In contrast, LPS responses were constrained at birth at which point they were largely restricted to monocytes and HSPCs. Moreover, we observed a division of labour in the LPS responses, where monocytes/HSPC upregulated proinflammatory molecules whereas lymphocyte populations elicited IRF1/STAT1-mediated IFN-signaling pathways. Importantly, these responses exhibited substantial intercellular crosstalk and markedly strengthened between birth and age 5 years. Finally, we observed distinct activation/response trajectory endpoints inferred for CBMC- and PBMC-derived monocytes stimulated with LPS, and this was not apparent among samples exposed to Poly(I:C). Despite the size of our study, these findings offer proof-of-principle feasibility to capture cell type-specific and context-specific gene regulatory programs that underlie innate immune function at birth versus age 5 and provides a framework for future studies to track innate immune function across early life in relation to environment exposures and disease risk.

From our gene expression analysis, we found that Poly(I:C) provoked a robust IFN-signalling response by all cell types detected, and this was relatively stable between birth and 5 years. In contrast, the LPS response was more constrained at birth compared to early childhood and, of the cell types detected in this study, proinflammatory responses were primarily mediated by CD14^+^ monocytes, alongside HSPCs, supporting their role as immune effectors (41). Strikingly, we observed a partitioning among immune cell types following LPS stimulation whereby archetypal proinflammatory genes (e.g., *IL1B*, *CXCL8*) were upregulated in CD14^+^ monocytes while IFN-signaling genes (e.g., *STAT1*) were upregulated in lymphocytes (T and B cells). Notably, the transcriptional response to LPS displayed more profound differences between birth and 5 years compared to matched samples exposed to Poly(I:C). At the level of gene regulatory networks, we found that IFN-related IRF1 and STAT1 transcription factors (42) were the master regulators of the lymphocyte response to LPS stimulation, in agreement with their corresponding gene expression, whereas the LPS-induced monocyte response was mediated by inflammatory regulators (e.g., FOS, JUN, ATF3) (43, 44). Furthermore, *in silico* perturbation (35) to simulate the blockade of IRF1, IRF7, and STAT1, reinforced the central control of IRF1 and STAT1 in LPS-induced IFN-signalling networks of CD4 T cells at birth, and demonstrated that the influence of IRF7, the quintessential driver of type I IFN responses (45), within this network is more pronounced at 5 years.

Immune responses are mediated by the activation of multiple cell populations that transition through dynamic molecular states. Employing pseudotime trajectory inference, we found that naïve CD4^+^ T cells from resting CBMC samples clustered separately, consistent with notion that neonatal T cells represent a distinct lineage of cells (2, 8). In contrast, LPS-induced monocyte activation trajectories started from a common baseline which subsequently diverged into age-specific end points. Monocytes trajectories from Poly(I:C)-induced CBMC/5yr PBMC responses exhibited a single trajectory, suggesting that the developmental regulation of innate immune function is much more profound for LPS responses compared with Poly(I:C) responses, as we have reported previously (46, 47). We also observed striking differences in cellular composition between CBMC/PBMC samples, such as the lack of MAIT cells at birth, which is presumably explained by the fact that MAIT cells are driven by bacterial metabolites (48, 49), and consequently develop in parallel with the infant microbiome (50).

Immune responses are governed by complex cellular interactions that are primarily mediated by ligand-receptor signals (51). We systematically inferred intercellular communication networks from ligand-receptor gene expression data which revealed substantial crosstalk between myeloid and lymphoid lineage cell types, indicating a coordinated response to LPS and Poly(I:C) stimulation. Additionally, we identified several age-specific interactions, such as extensive HSPC crosstalk in CBMC samples and less discriminate ligand binding to T cell surface receptors at birth. For example, molecular interactions were observed between LPS-induced monocyte ligands (e.g., IL6, CCL2/3/7) and naïve CD4^+^ T cell receptors (e.g., IL6R, CCR1/2/5) in CBMC samples that were not observed at age 5yrs. These temporally restricted monocyte-CD4^+^ T cell interactions in turn elicited STAT1 activity, suggesting that the mechanisms that determine IFN responses to LPS are qualitatively different at birth versus 5 years of age. Conversely, our analysis of naïve CD4^+^ T cell ligand interactions with monocyte receptors showed that regulatory activity for FOXO3 – which has been implicated in inflammation cytokine production and TLR4 upregulation specifically in LPS-induced monocytes (52) – was restricted to the 5-year LPS response, serving as an example of age-related differences in the regulation of LPS-induced monocyte responses that result in activation trajectory branching.

Our study has several limitations which we acknowledged, as follows. First, findings from our study are based on data collected from 12 scRNA-Seq samples generated from two biological donors, and accordingly follow-up studies in larger sample sizes are required to extend the findings to the general population. Second, we studied innate immune function at a single time point using two ligands. Future studies could investigate multiple time points and a broader panel of stimuli including intact viruses and bacteria. Additionally, we acknowledge that the immune system undergoes dynamic changes in the first week of life (53), and accordingly, innate immune responses in cord blood represent baseline responses at birth. Additionally, our study focused on circulating immune cells, although cells in the airway may promote additional layers of control in response to these stimuli (54). Finally, future studies employing whole blood samples could extend these finding to cell types not captured among the mononuclear cell compartment from the samples investigated in this study. Notwithstanding these limitations, our study highlights several cell type-, stimuli-, and age-specific features of innate function of immune cells in early life, including underlying gene expression response programs, gene regulatory networks, and patterns of intercellular communication. These findings are relevant to future studies designed to dissect these mechanisms in relation to environmental exposures and disease risk.

## Data availability statement

The datasets presented in this study can be found in online repositories. The names of the repository/repositories and accession number(s) can be found in the article/**Supplementary Material**.

## Ethics statement

The studies involving humans were approved by The University of Western Australia (reference RA/4/1/7560). The studies were conducted in accordance with the local legislation and institutional requirements. Written informed consent for participation in this study was provided by the participants’ legal guardians/next of kin.

## Author contributions

JR: Conceptualization, Data curation, Formal Analysis, Investigation, Methodology, Software, Visualization, Writing – original draft, Writing – review & editing. MS: Investigation, Methodology, Writing – review & editing. JA: Methodology, Resources, Writing – review & editing. MI: Investigation, Writing – review & editing. MC: Methodology, Resources, Writing – review & editing. AS: Resources, Writing – review & editing. DS: Methodology, Writing – review & editing. JW: Resources, Writing – review & editing. PH: Conceptualization, Data curation, Funding acquisition, Methodology, Project administration, Resources, Supervision, Writing – review & editing. AB: Conceptualization, Data curation, Funding acquisition, Investigation, Methodology, Project administration, Resources, Software, Supervision, Writing – original draft, Writing – review & editing.
