# Supplementary material for: Single cell transcriptomics reveals cell type specific features of developmentally regulated responses to lipopolysaccharide between birth and 5 years
Source: Front Immunol. 2023 Oct 17;14:1275937. doi: 10.3389/fimmu.2023.1275937 (PMC10619903; doi:10.3389/fimmu.2023.1275937)
Supplement: Supplementary file 1 [file DataSheet_1.pdf]

Table S1. Table of quality control metrics recorded for cell viability, CellRanger, and post-alignment quality control.

	Donor	Donor 1						Donor 1						
	Sample collection age	CBMC			5yr PBMC			CBMC			5yr PBMC			
	Condition	CTRL	LPS	Poly(I:C)	CTRL	LPS	Poly(I:C)	CTRL	LPS	Poly(I:C)	CTRL	LPS	Poly(I:C)	Average
	Post-thaw cell viability (%)	94.67			84.25			94.93			93.79			**91.91**
	Post-culture cell viability	95.2	95.2	95.9	90.7	93.2	96.9	91.8	87.5	95	97	97.7	94.3	**94.2**
CellRanger ouput	Estimated number of cells	4416	4868	4989	6134	6189	5884	6065	4258	5540	6179	5810	5994	**5527.17**
	Mean reads/cell	72730	68933	62540	58212	54895	52939	54043	88793	65676	63811	57225	57388	**63098.75**
	Estimated total UNI counts (millions)	33.8	39.2	33.7	37.3	36.3	29.7	29.7	29.9	35.9	34.7	31.4	31.8	**33.62**
	Mean UMI counts/cell	7658	8052	6763	6086	5861	5052	4893	7030	6486	5617	5400	5303	**6183.42**
	Median genes/cell	2276	2378	2252	1859	1869	1686	1700	2220	2177	1755	1772	1818	**1980.17**
	Total reads (millions)	321	335	312	357	339	311	327	378	363	394	332	343	**342.67**
	Valid barcodes (%)	96.8	97.1	97	96.9	96.9	96.5	97.5	96.6	97.1	97.7	97.3	97.4	**97.07**
	Valid UMIs (%)	99.9	99.9	99.9	99.9	99.9	100	99.9	99.9	99.9	100	100	100	**99.93**
	Reads mapped (%)	96.3	96.6	96.5	96.3	96.1	96.5	97.5	97.7	97.6	97.4	97.3	97.3	**96.93**
	Total unique genes detected	24802	24737	24308	24513	24432	23606	23903	24536	24729	24332	24257	24144	**24358.25**
Post-QC	Post-QC cells	3814	4250	4326	5321	4920	5108	5188	3632	4994	5774	5161	5420	**4825.7**
	Total counts (millions)	34.7	39.3	35.9	37.8	35.7	33.3	29.9	30.1	39	38.5	33.2	34.2	**35.1**
	Mean counts/cell	9104.7	9242	8291.2	7096.6	7267.3	6525.6	5764.9	8292.1	7801.6	6673.1	6440.9	6305.4	**7400.4**
	Mean genes detected/cell	2544.3	2573.4	2498.3	2088.6	2089.7	1972.5	1840.2	2440	2376.7	1944.5	1942.7	1972.7	**2190.3**
	Mean mitochondrial content (%)	8.3	7.7	7.6	8	7.3	7.5	8	7.7	7.1	8.2	8.3	7.3	**7.7**
	Total unique genes detected	19599	19203	18792	18500	18269	17472	17554	19359	19103	18150	17982	17801	**18482**

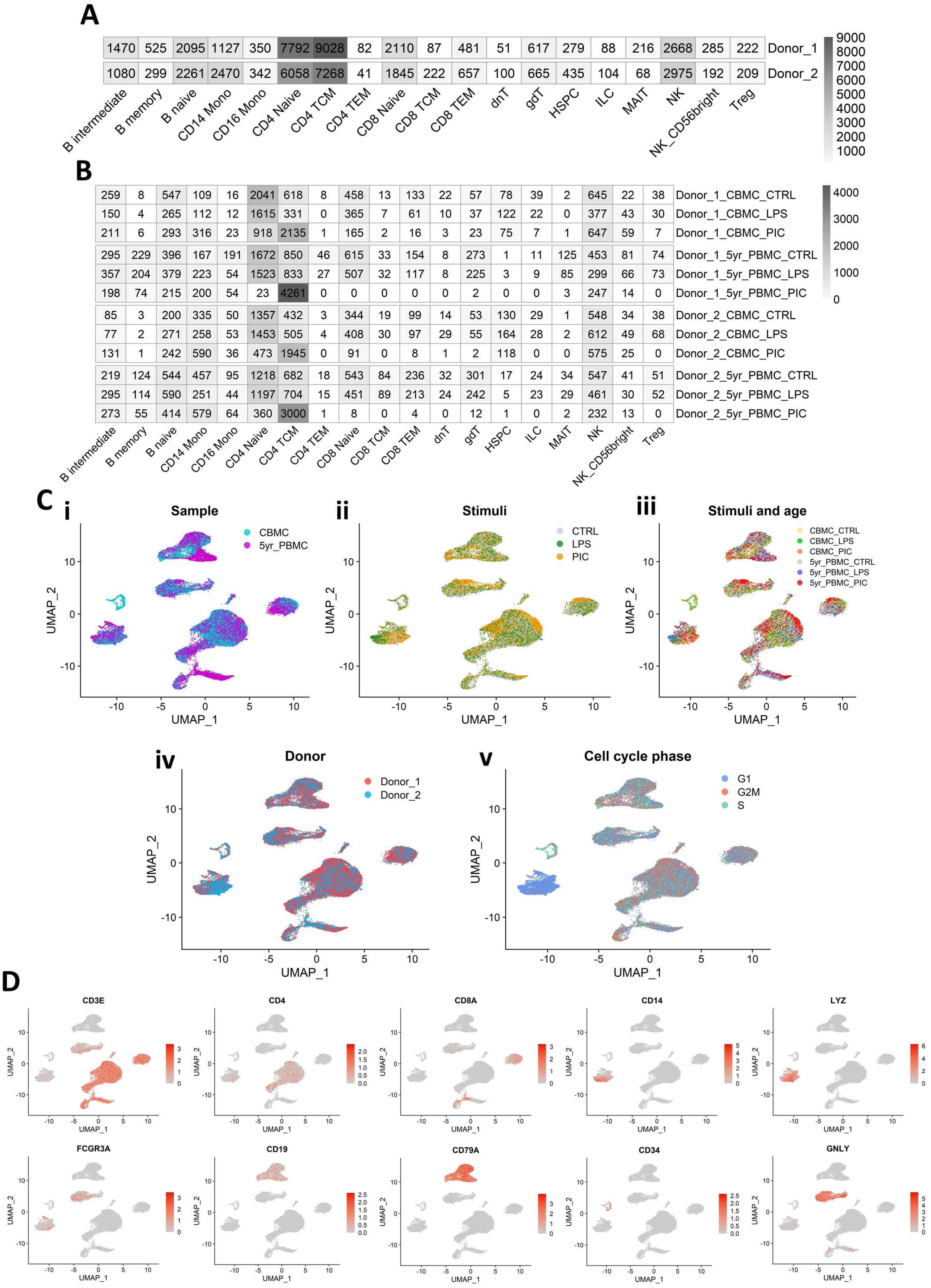
A
1470 525 2095 1127 350 7792 9028 82 2110 87 481 51 617 279 88 216 2668 285 222 Donor_1
1080 299 2261 2470 342 6058 7268 41 1845 222 657 100 665 435 104 68 2975 192 209 Donor_2
B intermediate
B memory
B naive
CD14 Mono
CD16 Mono
CD4 Naive
CD4 TCM
CD4 TEM
CD8 Naive
CD8 TCM
CD8 TEM
dnT
gdT
HSPC
ILC
MAIT
NK
NK_CD56bright
Treg
9000 8000 7000 6000 5000 4000 3000 2000 1000
B
259 8 547 109 16 2041 618 8 458 13 133 22 57 78 39 2 645 22 38 Donor_1_CBMC_CTRL
150 4 265 112 12 1615 331 0 365 7 61 10 37 122 22 0 377 43 30 Donor_1_CBMC_LPS
211 6 293 316 23 918 2135 1 165 2 16 3 23 75 7 1 647 59 7 Donor_1_CBMC_PIC
295 229 396 167 191 1672 850 46 615 33 154 8 273 1 11 125 453 81 74 Donor_1_5yr_PBMC_CTRL
357 204 379 223 54 1523 833 27 507 32 117 8 225 3 9 85 299 66 73 Donor_1_5yr_PBMC_LPS
198 74 215 200 54 23 4261 0 0 0 0 0 2 0 0 3 247 14 0 Donor_1_5yr_PBMC_PIC
85 3 200 335 50 1357 432 3 344 19 99 14 53 130 29 1 548 34 38 Donor_2_CBMC_CTRL
77 2 271 258 53 1453 505 4 408 30 97 29 55 164 28 2 612 49 68 Donor_2_CBMC_LPS
131 1 242 590 36 473 1945 0 91 0 8 1 2 118 0 0 575 25 0 Donor_2_CBMC_PIC
219 124 544 457 95 1218 682 18 543 84 236 32 301 17 24 34 547 41 51 Donor_2_5yr_PBMC_CTRL
295 114 590 251 44 1197 704 15 451 89 213 24 242 5 23 29 461 30 52 Donor_2_5yr_PBMC_LPS
273 55 414 579 64 360 3000 1 8 0 4 0 12 1 0 2 232 13 0 Donor_2_5yr_PBMC_PIC
B intermediate
B memory
B naive
CD14 Mono
CD16 Mono
CD4 Naive
CD4 TCM
CD4 TEM
CD8 Naive
CD8 TCM
CD8 TEM
dnT
gdT
HSPC
ILC
MAIT
NK
NK_CD56bright
Treg
4000 3000 2000 1000 0
C
i
Sample
CBMC
5yr_PBMC
ii
Stimuli
CTRL
LPS
PIC
iii
Stimuli and age
CBMC_CTRL
CBMC_LPS
CBMC_PIC
5yr_PBMC_CTRL
5yr_PBMC_LPS
5yr_PBMC_PIC
iv
Donor
Donor_1
Donor_2
v
Cell cycle phase
G1
G2M
S
UMAP_1
UMAP_2
D
CD3E
CD4
CD8A
CD14
LYZ
FCGR3A
CD19
CD79A
CD34
GNLY

Figure S1. Cell counts by Donor/group and additional UMAP plots overlayed with selected sample characteristics. **A**) Heatmap showing cell counts for each of the Azimuth annotated cell types stratified by the donor the cells were generated from. **B**) Heatmap showing cell counts for each of the Azimuth annotated cell types stratified by the donor, sample collection age, and stimuli received. **C**) UMAP plots (same in Figure 1B) stratified by sample collection time point (**i**), stimuli/age group (**ii**), Biological donor (**iii**), and cell cycle phase (**iv**). **D**) Integrated UMAPs overlayed with selected marker genes.

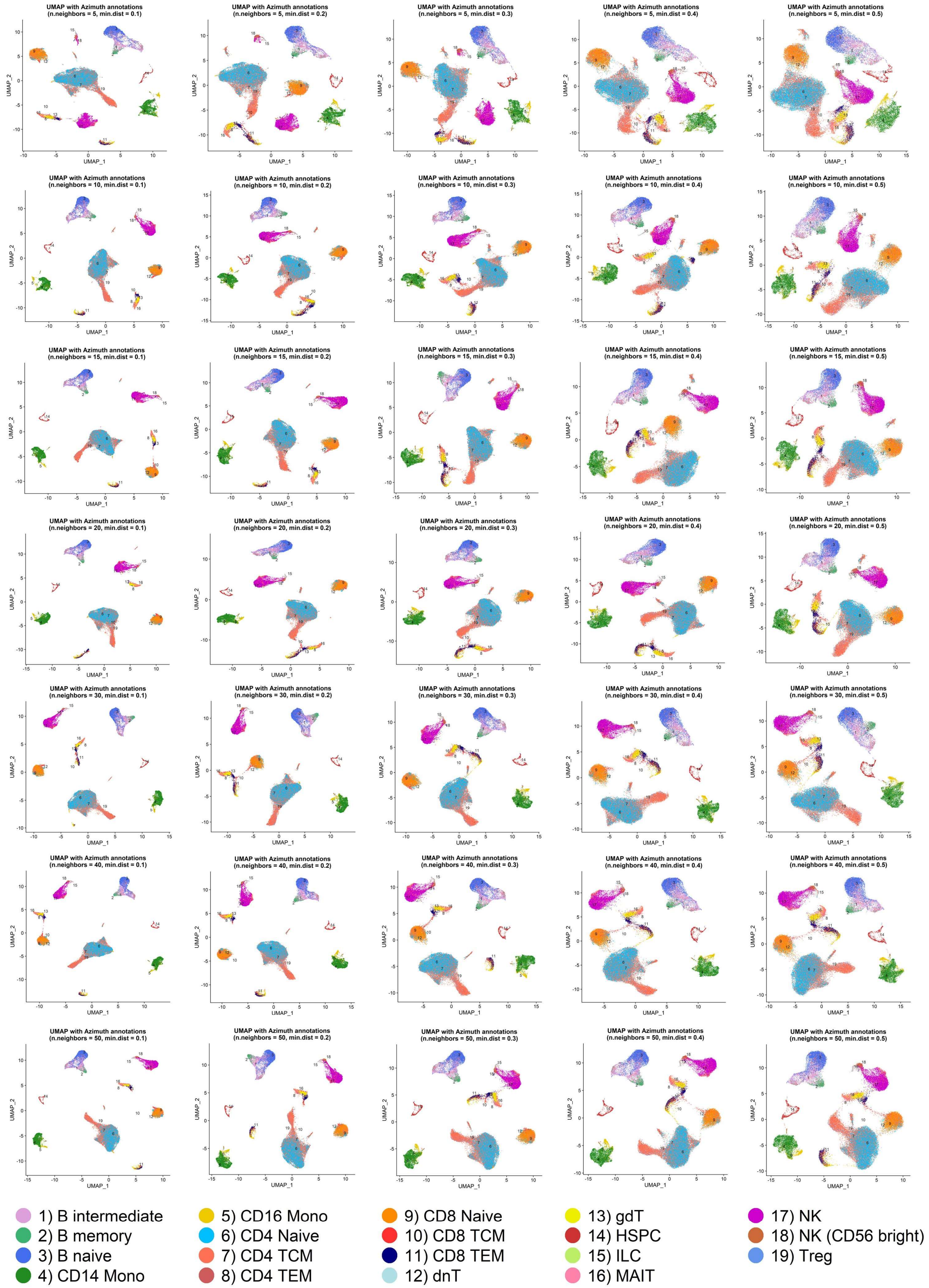

UMAP with Azimuth annotations (n.neighbors = 5, min.dist = 0.1)
UMAP with Azimuth annotations (n.neighbors = 5, min.dist = 0.2)
UMAP with Azimuth annotations (n.neighbors = 5, min.dist = 0.3)
UMAP with Azimuth annotations (n.neighbors = 5, min.dist = 0.4)
UMAP with Azimuth annotations (n.neighbors = 5, min.dist = 0.5)
UMAP with Azimuth annotations (n.neighbors = 10, min.dist = 0.1)
UMAP with Azimuth annotations (n.neighbors = 10, min.dist = 0.2)
UMAP with Azimuth annotations (n.neighbors = 10, min.dist = 0.3)
UMAP with Azimuth annotations (n.neighbors = 10, min.dist = 0.4)
UMAP with Azimuth annotations (n.neighbors = 10, min.dist = 0.5)
UMAP with Azimuth annotations (n.neighbors = 15, min.dist = 0.1)
UMAP with Azimuth annotations (n.neighbors = 15, min.dist = 0.2)
UMAP with Azimuth annotations (n.neighbors = 15, min.dist = 0.3)
UMAP with Azimuth annotations (n.neighbors = 15, min.dist = 0.4)
UMAP with Azimuth annotations (n.neighbors = 15, min.dist = 0.5)
UMAP with Azimuth annotations (n.neighbors = 20, min.dist = 0.1)
UMAP with Azimuth annotations (n.neighbors = 20, min.dist = 0.2)
UMAP with Azimuth annotations (n.neighbors = 20, min.dist = 0.3)
UMAP with Azimuth annotations (n.neighbors = 20, min.dist = 0.4)
UMAP with Azimuth annotations (n.neighbors = 20, min.dist = 0.5)
UMAP with Azimuth annotations (n.neighbors = 30, min.dist = 0.1)
UMAP with Azimuth annotations (n.neighbors = 30, min.dist = 0.2)
UMAP with Azimuth annotations (n.neighbors = 30, min.dist = 0.3)
UMAP with Azimuth annotations (n.neighbors = 30, min.dist = 0.4)
UMAP with Azimuth annotations (n.neighbors = 30, min.dist = 0.5)
UMAP with Azimuth annotations (n.neighbors = 40, min.dist = 0.1)
UMAP with Azimuth annotations (n.neighbors = 40, min.dist = 0.2)
UMAP with Azimuth annotations (n.neighbors = 40, min.dist = 0.3)
UMAP with Azimuth annotations (n.neighbors = 40, min.dist = 0.4)
UMAP with Azimuth annotations (n.neighbors = 40, min.dist = 0.5)
UMAP with Azimuth annotations (n.neighbors = 50, min.dist = 0.1)
UMAP with Azimuth annotations (n.neighbors = 50, min.dist = 0.2)
UMAP with Azimuth annotations (n.neighbors = 50, min.dist = 0.3)
UMAP with Azimuth annotations (n.neighbors = 50, min.dist = 0.4)
UMAP with Azimuth annotations (n.neighbors = 50, min.dist = 0.5)
UMAP_1
UMAP_2
1) B intermediate
2) B memory
3) B naive
4) CD14 Mono
5) CD16 Mono
6) CD4 Naive
7) CD4 TCM
8) CD4 TEM
9) CD8 Naive
10) CD8 TCM
11) CD8 TEM
12) dnT
13) gdT
14) HSPC
15) ILC
16) MAIT
17) NK
18) NK (CD56 bright)
19) Treg

Figure S2. Repeat UMAP with varying graph construction parameters. UMAP construction and plotting was repeated with different values for the number of nearest neighbors (n.neighbors) and minimum distance (min.dist). Plots with increasing minimum distance are shown from left to right and increasing number of nearest neighbors from top to bottom. The values for minimum distance are 0.1, 0.2, 0.3, 0.4, and 0.5, and the values for number of nearest neighbors are 5, 10, 15, 20, 30, 40, and 50. Each plot is overlayered with the Azimuth reference-based annotation for each cell, with a common legend at the bottom.

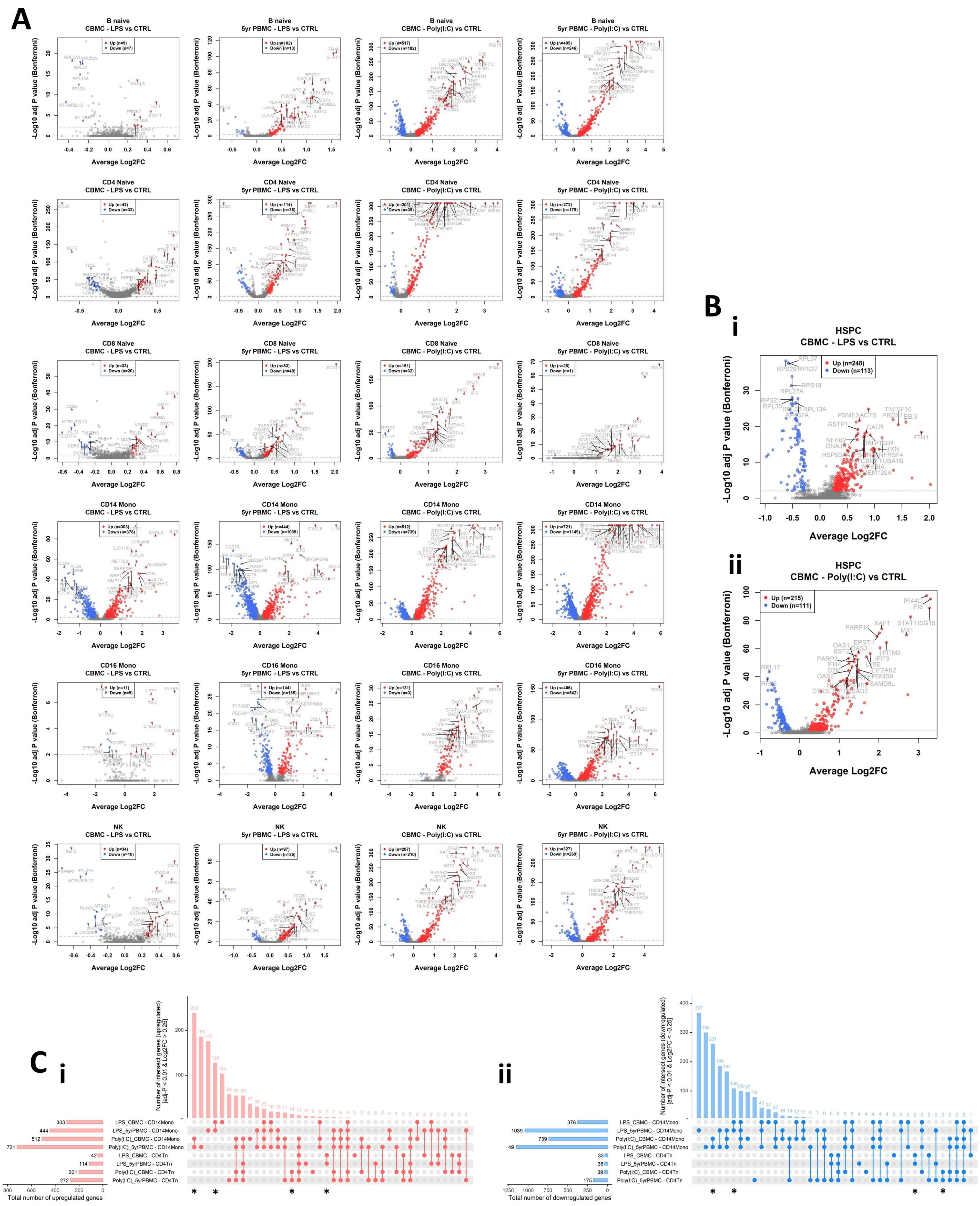
A
B naive
CBMC - LPS vs CTRL
5yr PBMC - LPS vs CTRL
CBMC - Poly(I:C) vs CTRL
5yr PBMC - Poly(I:C) vs CTRL
CD4 Naive
CD8 Naive
CD14 Mono
CD16 Mono
NK
-Log10 adj P value (Bonferroni)
Average Log2FC
B
i
HSPC
CBMC - LPS vs CTRL
Up (n=248)
Down (n=113)
ii
HSPC
CBMC - Poly(I:C) vs CTRL
Up (n=215)
Down (n=111)
C
i
Number of intersect genes (upregulated)
[adj-P < 0.01 & Log2FC > 0.25]
LPS_CBMC - CD14Mono
LPS_5yrPBMC - CD14Mono
Poly(I:C)_CBMC - CD14Mono
Poly(I:C)_5yrPBMC - CD14Mono
LPS_CBMC - CD4Tn
LPS_5yrPBMC - CD4Tn
Poly(I:C)_CBMC - CD4Tn
Poly(I:C)_5yrPBMC - CD4Tn
Total number of upregulated genes
ii
Number of intersect genes (downregulated)
[adj-P < 0.01 & Log2FC < -0.25]
Total number of downregulated genes

Figure S3. **A**) Volcano plots of selected comparisons from the differential expression analysis. The plots are arranged to show the results for Stimuli compared to unstimulated controls for the LPS-stimulated CBMC (first column), LPS-stimulated 5-year PBMC (second column), Poly(I:C)-stimulated CBMC (third column), and Poly(I:C)-stimulated 5-year PBMC (fourth column). Each row corresponds to a different cell type (naïve B cells, naïve $CD4^+$ T cells, naïve $CD8^+$ T cells $CD14^+$ Monocytes, $CD16^+$ Monocytes, and NK cells). Each point represents a gene, and the x-axis shows the average Log_2-fold change, and the y-axis shows the -Log_{10} Bonferroni-corrected *p* value for the corresponding comparison. Genes which recorded a corrected *p* value < 0.01 and an absolute average log_2-FC > 0.25 were considered significantly dysregulated and are shown as red (upregulated) and blue (downregulated) points. The total number of up- and down-regulated genes are shown in the inset of each plot, with selected dysregulated genes annotated. **B**) Volcano plots differentially expressed genes for CBMC HSPCs stimulated with LPS (**i**) and Poly(I:C) (**ii**) compared to controls. Plot parameters are the same as above. **C**) Upset plots showing the overlap of significantly upregulated (**i**) and downregulated (**ii**) genes between $CD14^+$ Monocytes and naïve $CD4^+$ T cells from CBMC and 5yr PBMC samples stimulated with LPS and Poly(I:C). In each plot, the horizontal bar plot shows the total number of up/downregulated genes for that comparison and the vertical bar plot displays the number of intersect genes for each comparison denoted by the combination matrix. Intersects between CBMC and 5yr PBMC results for the same cell type/stimuli are indicated by a black asterix.

A

	Donor_1	Donor_2
CBMC - CTRL	94	118
CBMC - LPS	100	113
CBMC - Poly(I:C)	286	251
5yr_PBMC - CTRL	151	185
5yr_PBMC - LPS	192	115
5yr_PBMC - Poly(I:C)	177	218
Total	1000	1000

B

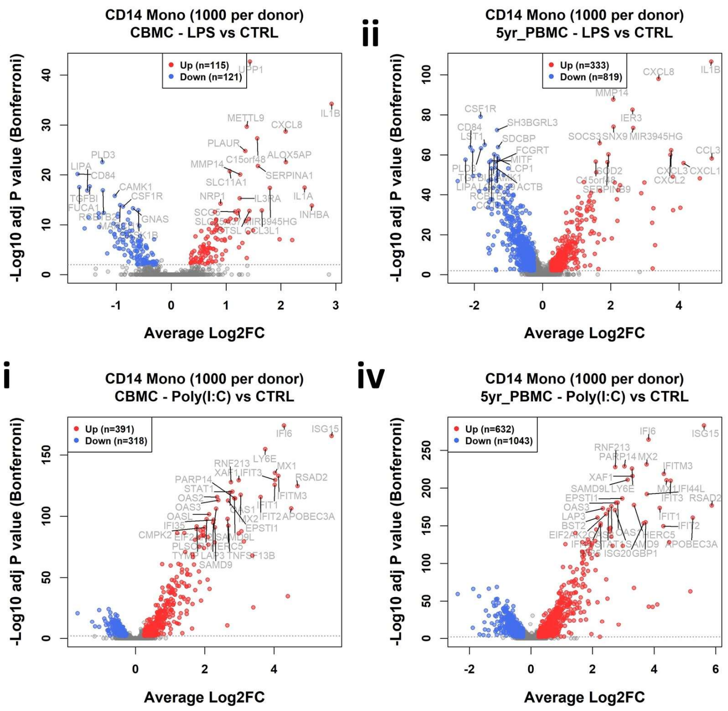

C

	Number of top dysregulated genes						
	10	50	100	200	500	750	1000
CBMC - LPS	90%	76%	86%	82.8%	80.2%	81.9%	83.1%
CBMC - Poly(I:C)	80%	100%	97%	90.8%	90.2%	88.7%	88.7%
5yr PBMC - LPS	80%	88%	90%	92.4%	90.8%	89.5%	89.7%
5yr PBMC - Poly(I:C)	20%	98%	96%	91.6%	90.8%	88.4%	89.4%

D

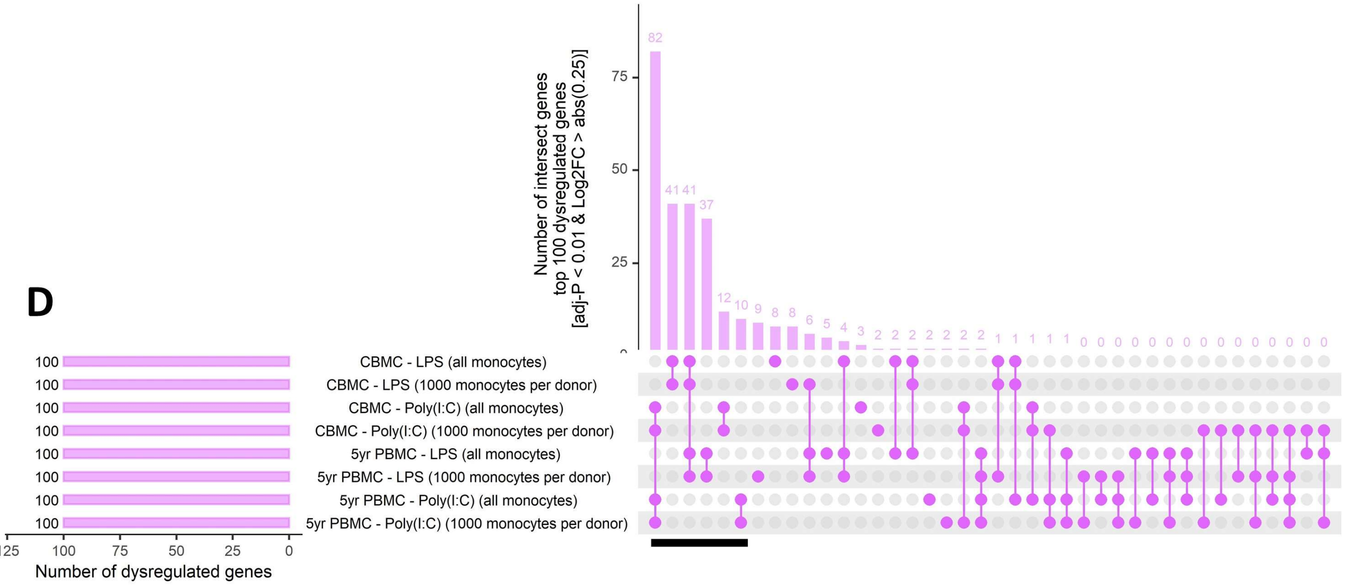

Figure S4. Results from the sub-analysis of $CD14^+$ monocytes which were randomly down-sampled to 1,000 cells per donor. **A**) Breakdown of the 1,000 cells per donor into sample/stimuli groups. **B**) Volcano plots of differentially expressed genes compared to unstimulated control for CD14+ monocytes from LPS-treated CBMC (**i**) and 5yr PBMC (**ii**) samples, and Poly(I:C)-treated CBMC (**iii**) and 5yr PBMC (**iv**) samples. The x-axis shows the average $\log_2$ fold change and the y-axis shows the $-\text{Log}_{10}$ Bonferroni-corrected *p* value. The dashed grey line indicates a Bonferroni-corrected *p* value of 0.01. Points colored red and blue represent gene with are considered significantly upregulated and downregulated, respectively. **C**) Tabulated percentage overlap of the top dysregulated genes (ordered by adjusted-*p* value) between differential expression analysis results from all CD14+ monocytes and the randomly selected CD14+ monocyte subset. The columns represent the number of top gene selected and the row show the comparison. As an example, for the comparison of LPS versus control in 5-year PBMC samples (third row), 89.7% of the top 1000 DEGs were common to both the analysis of all monocytes and the analysis of the randomly selected $CD14^+$ monocyte subset. **D**) Upset plots showing the overlap of the top 100 significantly upregulated genes for CBMC- and 5yr PBMC-derived $CD14^+$ monocytes stimulated with LPS and Poly(I:C). The horizontal bar plot shows the total number of upregulated genes considered (here, 100 top genes) for each comparison. The 100 DEGs identified from the analyses of the randomly selected $CD14^+$ monocyte subset are denoted as '1000 monocytes per donor'. The vertical bar plot displays the number of intersect genes for each comparison indicated in the combination matrix. The black bar below the combination matrix indicates prominent intersect sets which involve matching comparisons between the 'all' and '1,000 per donor' $CD14^+$ monocyte analyses.

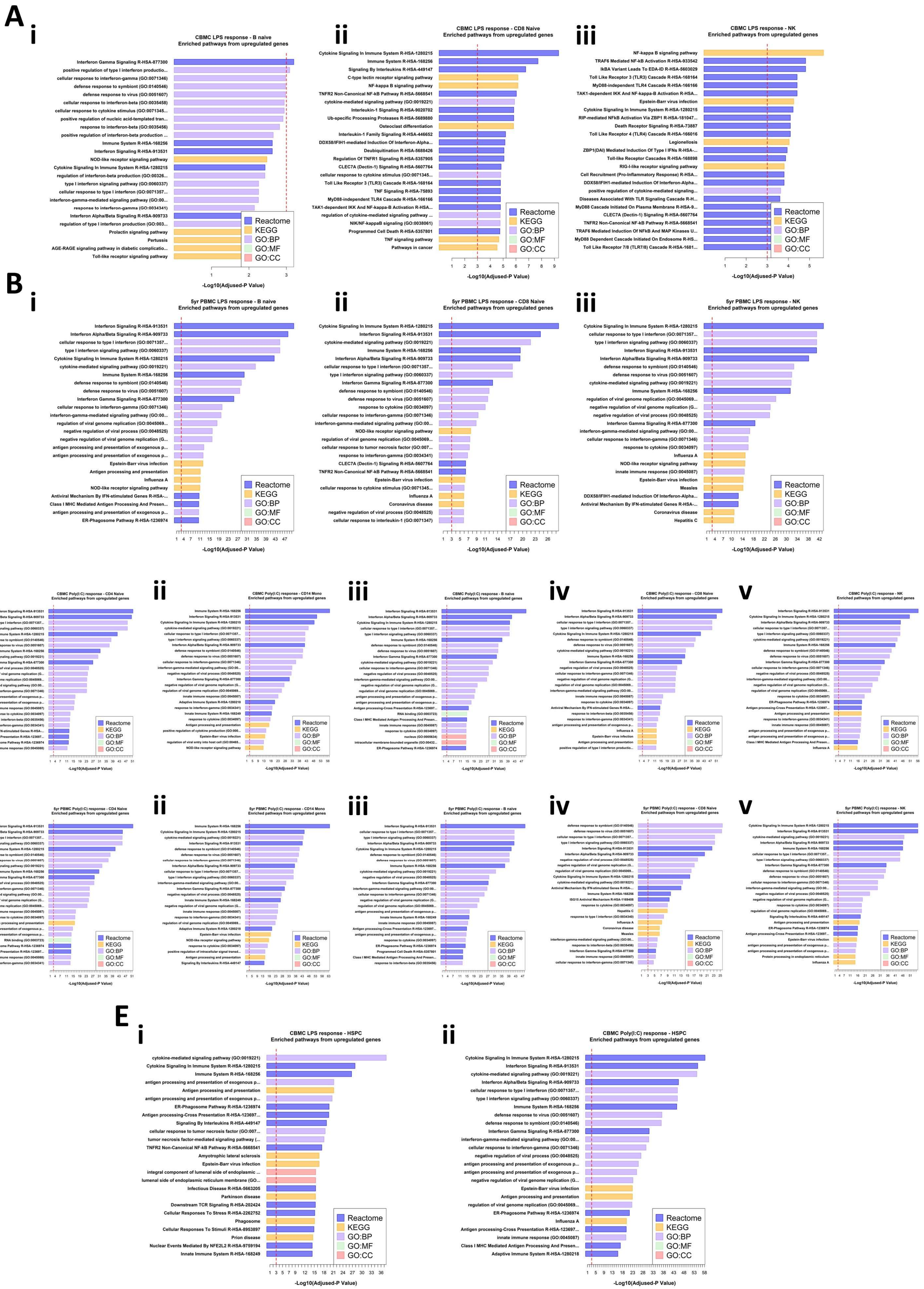
A
i
CBMC LPS response - B naive
Enriched pathways from upregulated genes
Interferon Gamma Signaling R-HSA-877300
positive regulation of type I interferon productio...
cellular response to interferon-gamma (GO:0071346)
defense response to symbiont (GO:0140546)
defense response to virus (GO:0051607)
cellular response to interferon-beta (GO:0035458)
cellular response to cytokine stimulus (GO:0071345...
positive regulation of nucleic acid-templated tran...
response to interferon-beta (GO:0035456)
positive regulation of interferon-beta production ...
Immune System R-HSA-168256
Interferon Signaling R-HSA-913531
NOD-like receptor signaling pathway
Cytokine Signaling In Immune System R-HSA-1280215
regulation of interferon-beta production (GO:00326...
type I interferon signaling pathway (GO:0060337)
cellular response to type I interferon (GO:0071357...
interferon-gamma-mediated signaling pathway (GO:00...
response to interferon-gamma (GO:0034341)
Interferon Alpha/Beta Signaling R-HSA-909733
regulation of type I interferon production (GO:003...
Prolactin signaling pathway
Pertussis
AGE-RAGE signaling pathway in diabetic complicatio...
Toll-like receptor signaling pathway
Reactome
KEGG
GO:BP
GO:MF
GO:CC
-Log10(Adjused-P Value)
ii
CBMC LPS response - CD8 Naive
Enriched pathways from upregulated genes
Cytokine Signaling In Immune System R-HSA-1280215
Immune System R-HSA-168256
Signaling By Interleukins R-HSA-449147
C-type lectin receptor signaling pathway
NF-kappa B signaling pathway
TNFR2 Non-Canonical NF-kB Pathway R-HSA-5668541
cytokine-mediated signaling pathway (GO:0019221)
Interleukin-1 Signaling R-HSA-9020702
Ub-specific Processing Proteases R-HSA-5689880
Osteoclast differentiation
Interleukin-1 Family Signaling R-HSA-446652
DDX58/IFIH1-mediated Induction Of Interferon-Alpha...
Deubiquitination R-HSA-5688426
Regulation Of TNFR1 Signaling R-HSA-5357905
CLEC7A (Dectin-1) Signaling R-HSA-5607764
cellular response to cytokine stimulus (GO:0071345...
Toll Like Receptor 3 (TLR3) Cascade R-HSA-168164
TNF Signaling R-HSA-75893
MyD88-independent TLR4 Cascade R-HSA-166166
TAK1-dependent IKK and NF-kappa-B Activation R-HSA...
regulation of cytokine-mediated signaling pathway ...
NIK/NF-kappaB signaling (GO:0038061)
Programmed Cell Death R-HSA-5357801
TNF signaling pathway
Pathways in cancer
-Log10(Adjused-P Value)
iii
CBMC LPS response - NK
Enriched pathways from upregulated genes
NF-kappa B signaling pathway
TRAF6 Mediated NF-kB Activation R-HSA-933542
IkBA Variant Leads To EDA-ID R-HSA-5603029
Toll Like Receptor 3 (TLR3) Cascade R-HSA-168164
MyD88-independent TLR4 Cascade R-HSA-166166
TAK1-dependent IKK and NF-kappa-B Activation R-HSA...
Epstein-Barr virus infection
Cytokine Signaling In Immune System R-HSA-1280215
RIP-mediated NFkB Activation Via ZBP1 R-HSA-181047...
Death Receptor Signaling R-HSA-73887
Toll Like Receptor 4 (TLR4) Cascade R-HSA-166016
Legionellosis
ZBP1(DAI) Mediated Induction Of Type I IFNs R-HSA-...
Toll-like Receptor Cascades R-HSA-168898
RIG-I-like receptor signaling pathway
Cell Recruitment (Pro-Inflammatory Response) R-HSA...
DDX58/IFIH1-mediated Induction Of Interferon-Alpha...
positive regulation of cytokine-mediated signaling...
Diseases Associated With TLR Signaling Cascade R-H...
MyD88 Cascade Initiated On Plasma Membrane R-HSA-9...
CLEC7A (Dectin-1) Signaling R-HSA-5607764
TNFR2 Non-Canonical NF-kB Pathway R-HSA-5668541
TRAF6 Mediated Induction Of NFkB And MAP Kinases U...
MyD88 Dependent Cascade Initiated On Endosome R-HS...
Toll Like Receptor 7/8 (TLR7/8) Cascade R-HSA-1681...
-Log10(Adjused-P Value)
B
i
5yr PBMC LPS response - B naive
Enriched pathways from upregulated genes
Interferon Signaling R-HSA-913531
Interferon Alpha/Beta Signaling R-HSA-909733
cellular response to type I interferon (GO:0071357...
type I interferon signaling pathway (GO:0060337)
Cytokine Signaling In Immune System R-HSA-1280215
cytokine-mediated signaling pathway (GO:0019221)
Immune System R-HSA-168256
defense response to symbiont (GO:0140546)
defense response to virus (GO:0051607)
Interferon Gamma Signaling R-HSA-877300
cellular response to interferon-gamma (GO:0071346)
interferon-gamma-mediated signaling pathway (GO:00...
regulation of viral genome replication (GO:0045069...
negative regulation of viral process (GO:0048525)
negative regulation of viral genome replication (G...
antigen processing and presentation of exogenous p...
antigen processing and presentation of exogenous p...
Epstein-Barr virus infection
Antigen processing and presentation
Influenza A
NOD-like receptor signaling pathway
Antiviral Mechanism By IFN-stimulated Genes R-HSA-...
Class I MHC Mediated Antigen Processing And Presen...
antigen processing and presentation of exogenous p...
ER-Phagosome Pathway R-HSA-1236974
-Log10(Adjused-P Value)
ii
5yr PBMC LPS response - CD8 Naive
Enriched pathways from upregulated genes
Cytokine Signaling In Immune System R-HSA-1280215
Interferon Signaling R-HSA-913531
cytokine-mediated signaling pathway (GO:0019221)
Immune System R-HSA-168256
Interferon Alpha/Beta Signaling R-HSA-909733
cellular response to type I interferon (GO:0071357...
type I interferon signaling pathway (GO:0060337)
Interferon Gamma Signaling R-HSA-877300
defense response to symbiont (GO:0140546)
defense response to virus (GO:0051607)
response to cytokine (GO:0034097)
cellular response to interferon-gamma (GO:0071346)
interferon-gamma-mediated signaling pathway (GO:00...
NOD-like receptor signaling pathway
regulation of viral genome replication (GO:0045069...
cellular response to tumor necrosis factor (GO:007...
response to interferon-gamma (GO:0034341)
CLEC7A (Dectin-1) Signaling R-HSA-5607764
TNFR2 Non-Canonical NF-kB Pathway R-HSA-5668541
Epstein-Barr virus infection
cellular response to cytokine stimulus (GO:0071345...
Influenza A
Coronavirus disease
negative regulation of viral process (GO:0048525)
cellular response to interleukin-1 (GO:0071347)
-Log10(Adjused-P Value)
iii
5yr PBMC LPS response - NK
Enriched pathways from upregulated genes
Cytokine Signaling In Immune System R-HSA-1280215
cellular response to type I interferon (GO:0071357...
type I interferon signaling pathway (GO:0060337)
Interferon Signaling R-HSA-913531
Interferon Alpha/Beta Signaling R-HSA-909733
defense response to symbiont (GO:0140546)
defense response to virus (GO:0051607)
cytokine-mediated signaling pathway (GO:0019221)
Immune System R-HSA-168256
regulation of viral genome replication (GO:0045069...
negative regulation of viral genome replication (G...
negative regulation of viral process (GO:0048525)
Interferon Gamma Signaling R-HSA-877300
interferon-gamma-mediated signaling pathway (GO:00...
cellular response to interferon-gamma (GO:0071346)
response to cytokine (GO:0034097)
Influenza A
NOD-like receptor signaling pathway
innate immune response (GO:0045087)
Epstein-Barr virus infection
Measles
DDX58/IFIH1-mediated Induction Of Interferon-Alpha...
Antiviral Mechanism By IFN-stimulated Genes R-HSA-...
Coronavirus disease
Hepatitis C
-Log10(Adjused-P Value)
C
i
ii
iii
iv
v
D
i
ii
iii
iv
v
E
i
CBMC LPS response - HSPC
Enriched pathways from upregulated genes
cytokine-mediated signaling pathway (GO:0019221)
Cytokine Signaling In Immune System R-HSA-1280215
Immune System R-HSA-168256
antigen processing and presentation of exogenous p...
Antigen processing and presentation
antigen processing and presentation of exogenous p...
ER-Phagosome Pathway R-HSA-1236974
Antigen processing-Cross Presentation R-HSA-123697...
Signaling By Interleukins R-HSA-449147
cellular response to tumor necrosis factor (GO:007...
tumor necrosis factor-mediated signaling pathway (...
TNFR2 Non-Canonical NF-kB Pathway R-HSA-5668541
Amyotrophic lateral sclerosis
Epstein-Barr virus infection
integral component of lumenal side of endoplasmic ...
lumenal side of endoplasmic reticulum membrane (GO...
Infectious Disease R-HSA-5663205
Parkinson disease
Downstream TCR Signaling R-HSA-202424
Cellular Responses To Stress R-HSA-2262752
Phagosome
Cellular Responses To Stimuli R-HSA-8953897
Prion disease
Nuclear Events Mediated By NFE2L2 R-HSA-9759194
Innate Immune System R-HSA-168249
Reactome
KEGG
GO:BP
GO:MF
GO:CC
-Log10(Adjused-P Value)
ii
CBMC Poly(I:C) response - HSPC
Enriched pathways from upregulated genes
Cytokine Signaling In Immune System R-HSA-1280215
Interferon Signaling R-HSA-913531
cytokine-mediated signaling pathway (GO:0019221)
Interferon Alpha/Beta Signaling R-HSA-909733
cellular response to type I interferon (GO:0071357...
type I interferon signaling pathway (GO:0060337)
Immune System R-HSA-168256
defense response to virus (GO:0051607)
defense response to symbiont (GO:0140546)
Interferon Gamma Signaling R-HSA-877300
interferon-gamma-mediated signaling pathway (GO:00...
cellular response to interferon-gamma (GO:0071346)
negative regulation of viral process (GO:0048525)
antigen processing and presentation of exogenous p...
antigen processing and presentation of exogenous p...
negative regulation of viral genome replication (G...
Epstein-Barr virus infection
Antigen processing and presentation
regulation of viral genome replication (GO:0045069...
ER-Phagosome Pathway R-HSA-1236974
Influenza A
Antigen processing-Cross Presentation R-HSA-123697...
innate immune response (GO:0045087)
Class I MHC Mediated Antigen Processing And Presen...
Adaptive Immune System R-HSA-1280218
-Log10(Adjused-P Value)

Figure S5. Pathways analysis of upregulated genes identified by differential expression analysis for selected cell types. **A**) Horizontal bar plots of the top 25 significantly enriched pathways from upregulated genes following LPS stimulation of CBMC samples compared to matched control. Results are shown for naïve B cells (**i**), naïve $CD8^+$ T cells (**ii**), and NK cells (**iii**). **B**) Same as (A) for 5-year PBMC samples treated with LPS compared to match control. Results are shown for naïve B cells (**i**), naïve $CD8^+$ T cells (**ii**), and NK cells (**iii**). **C**) Horizontal bar plots of the top 25 significantly enriched pathways from upregulated genes following Poly(I:C) stimulation of CBMC samples compared to matched control. Results are shown for naïve $CD4^+$ T cells (**i**), $CD14^+$ Monocytes (**ii**), naïve B cells (**iii**), naïve $CD8^+$ T cells (**iv**), and NK cells (**v**). **D**) Same as (C) for 5-year PBMC sample treated with Poly(I:C) compared to match control. **E**) Horizontal bar plots of the top 25 significantly enriched pathways from upregulated genes following LPS (**i**) and Poly(I:C) (**ii**) stimulation of CBMC samples compared to matched control. Foe reach plot, the x-axis shows the $-Log_{10}$ adjusted-P value associated with pathways enrichment, the dashed red line indicates an adjusted-*p* value of 0.001. Results are ordered from top by decreasing adjusted-*p* value for significantly enriched pathways identified from the Reactome, KEGG, and Gene Ontology (GO) databases. BP, Biological Process; MF, Molecular Function; CC, Cellular Compartment. See **supplementary data** for complete lists of significantly enriched pathways from dysregulated genes for all cell types analysed.

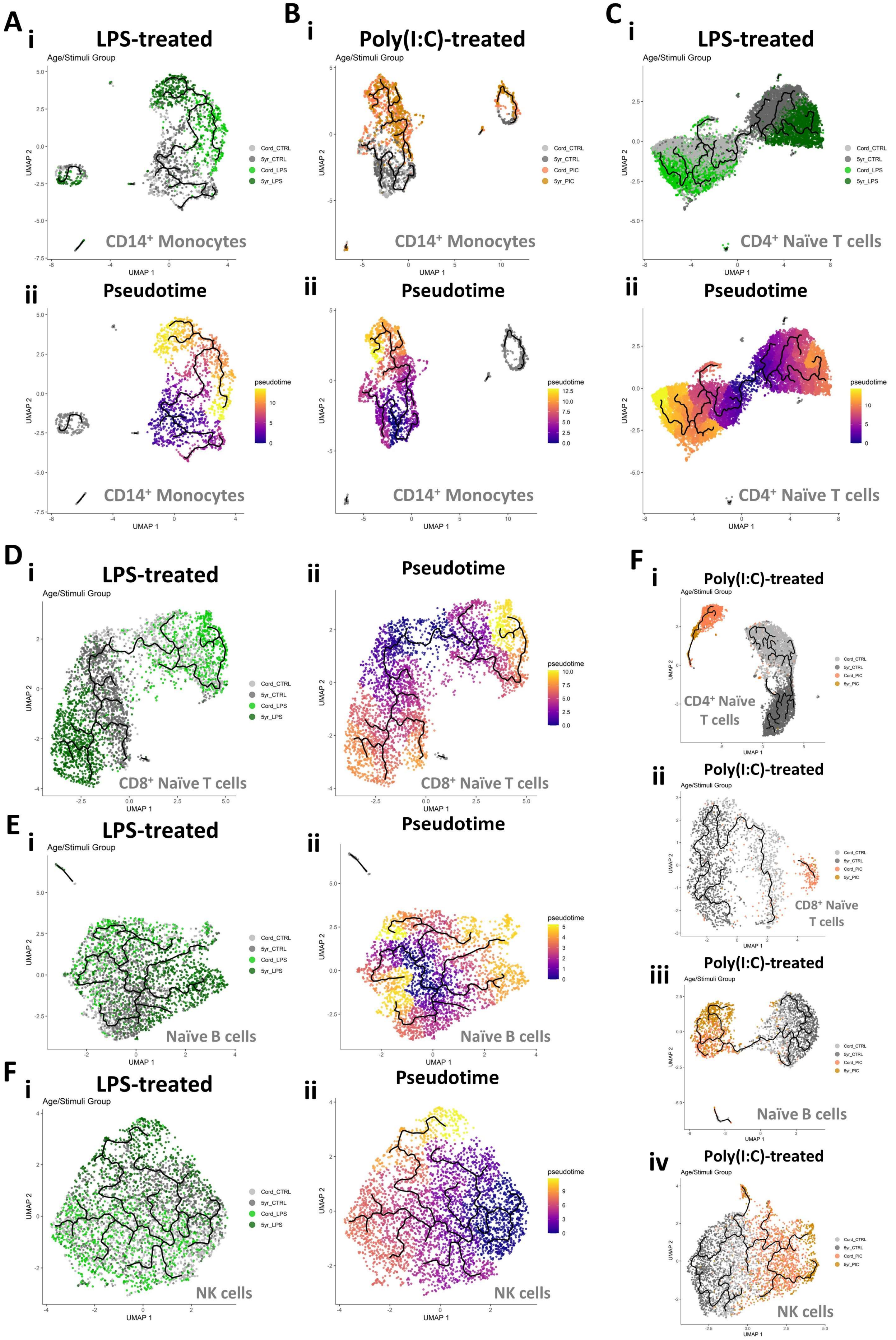
A
i
LPS-treated
Age/Stimuli Group
Cord_CTRL
5yr_CTRL
Cord_LPS
5yr_LPS
CD14⁺ Monocytes
UMAP 1
UMAP 2
ii
Pseudotime
pseudotime
CD14⁺ Monocytes
B
i
Poly(I:C)-treated
Age/Stimuli Group
Cord_CTRL
5yr_CTRL
Cord_PIC
5yr_PIC
CD14⁺ Monocytes
ii
Pseudotime
pseudotime
CD14⁺ Monocytes
C
i
LPS-treated
Age/Stimuli Group
CD4⁺ Naïve T cells
ii
Pseudotime
pseudotime
CD4⁺ Naïve T cells
D
i
LPS-treated
Age/Stimuli Group
CD8⁺ Naïve T cells
ii
Pseudotime
pseudotime
CD8⁺ Naïve T cells
E
i
LPS-treated
Age/Stimuli Group
Naïve B cells
ii
Pseudotime
pseudotime
Naïve B cells
F
i
LPS-treated
Age/Stimuli Group
NK cells
ii
Pseudotime
pseudotime
NK cells
F
i
Poly(I:C)-treated
Age/Stimuli Group
CD4⁺ Naïve T cells
ii
Poly(I:C)-treated
Age/Stimuli Group
CD8⁺ Naïve T cells
iii
Poly(I:C)-treated
Age/Stimuli Group
Naïve B cells
iv
Poly(I:C)-treated
Age/Stimuli Group
NK cells

Figure S6. Pseudotime trajectory mapping of cellular activation trajectories. **A-C**) UMAP plots representing cellular activation trajectories for LPS-stimulated CD14$^+$ monocytes (**A**), Poly(I:C)-stimulated CD14$^+$ monocytes (**B**), and LPS-stimulated CD4$^+$ naïve T cells (**C**). The first plot (**i**) in each panel is stratified by sample group and the second plot (**ii**) depicts the pseudotime. The branching black line on each plot represents the activation trajectory fitted to the data (monocle3 (33)). **D-F**) Plots from Monocle3 pseudotime analysis of additional cell types for samples stimulated with LPS and unstimulated controls. Plots are shown for CD8$^+$ naïve T cells (**D**), naïve B cells (**E**), and NK cells (**F**), with the first plot (**i**) in each panel is stratified by sample group and the second plot (**ii**) depicts the pseudotime. The branching black line on each plot represents the activation trajectory. **F**) UMAP plots from Monocle3 analysis of Poly(I:C)-stimulated CD4$^+$ naïve T cells (**i**), CD8$^+$ naïve T cells (**ii**), naïve B cells (**iii**), and NK cells (**iv**).

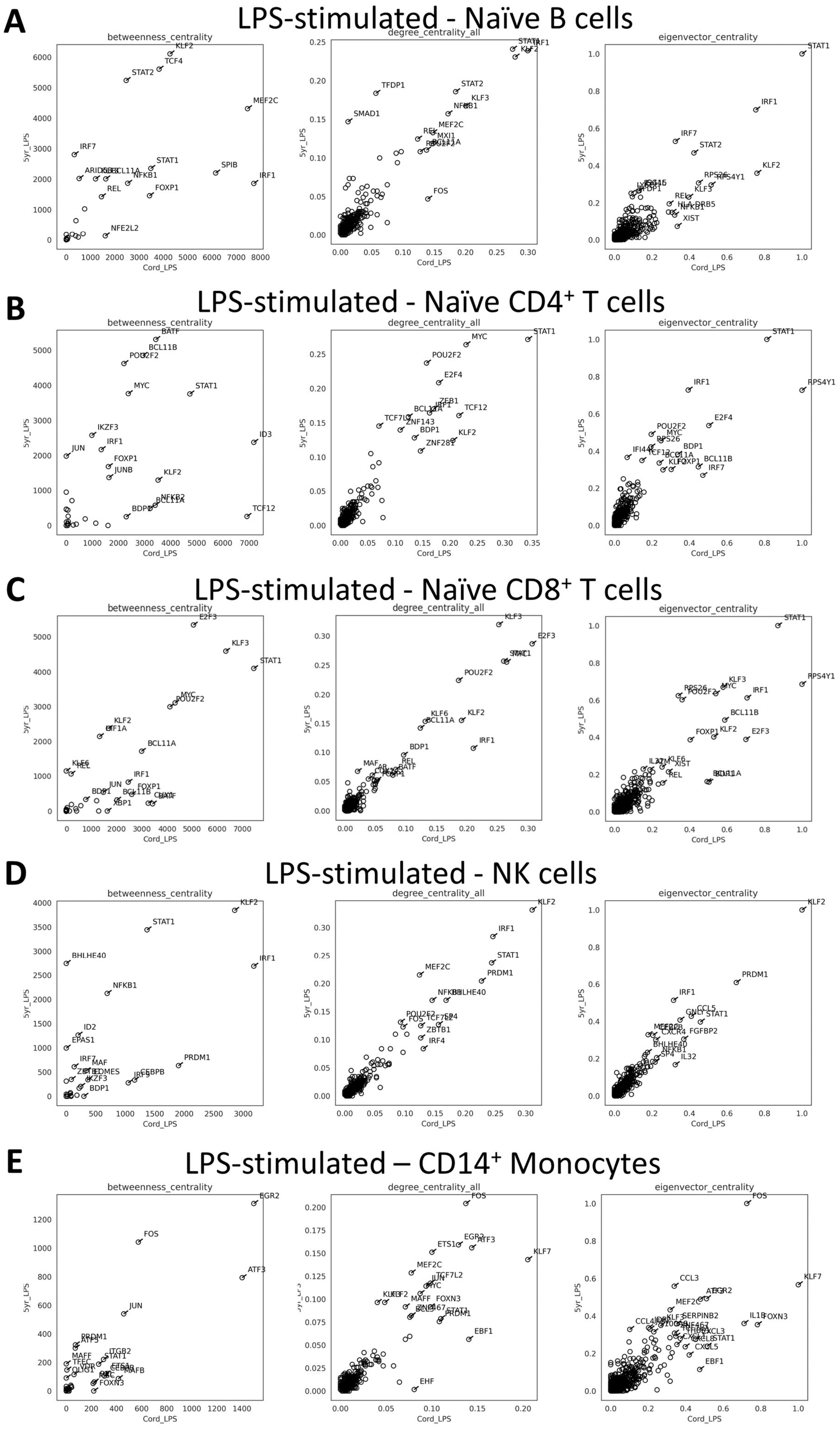
A
LPS-stimulated - Naïve B cells
B
LPS-stimulated - Naïve CD4+ T cells
C
LPS-stimulated - Naïve CD8+ T cells
D
LPS-stimulated - NK cells
E
LPS-stimulated – CD14+ Monocytes
betweenness_centrality
degree_centrality_all
eigenvector_centrality
Cord_LPS
5yr_LPS

Figure S7. Gene Regulatory Network characteristics for selected LPS-induced cell types. Plots show the correlation between the metrics for transcription factors and target genes for samples collected from cord blood (CBMC) (x-axis) and 5yr blood (PBMC) (y-axis). Plot are included for naïve B cells (**A**), naïve $CD4^+$ T cells (**B**), naïve $CD8^+$ T cells (**C**), NK cells (**D**), and $CD14^+$ monocytes (**E**), and show the betweenness centrality (left), degree centrality (middle), and eigenvector centrality (right). In each case, a higher value indicates a greater importance within the network and a deviation off the diagonal indicates the metric has a higher value in the corresponding sample.

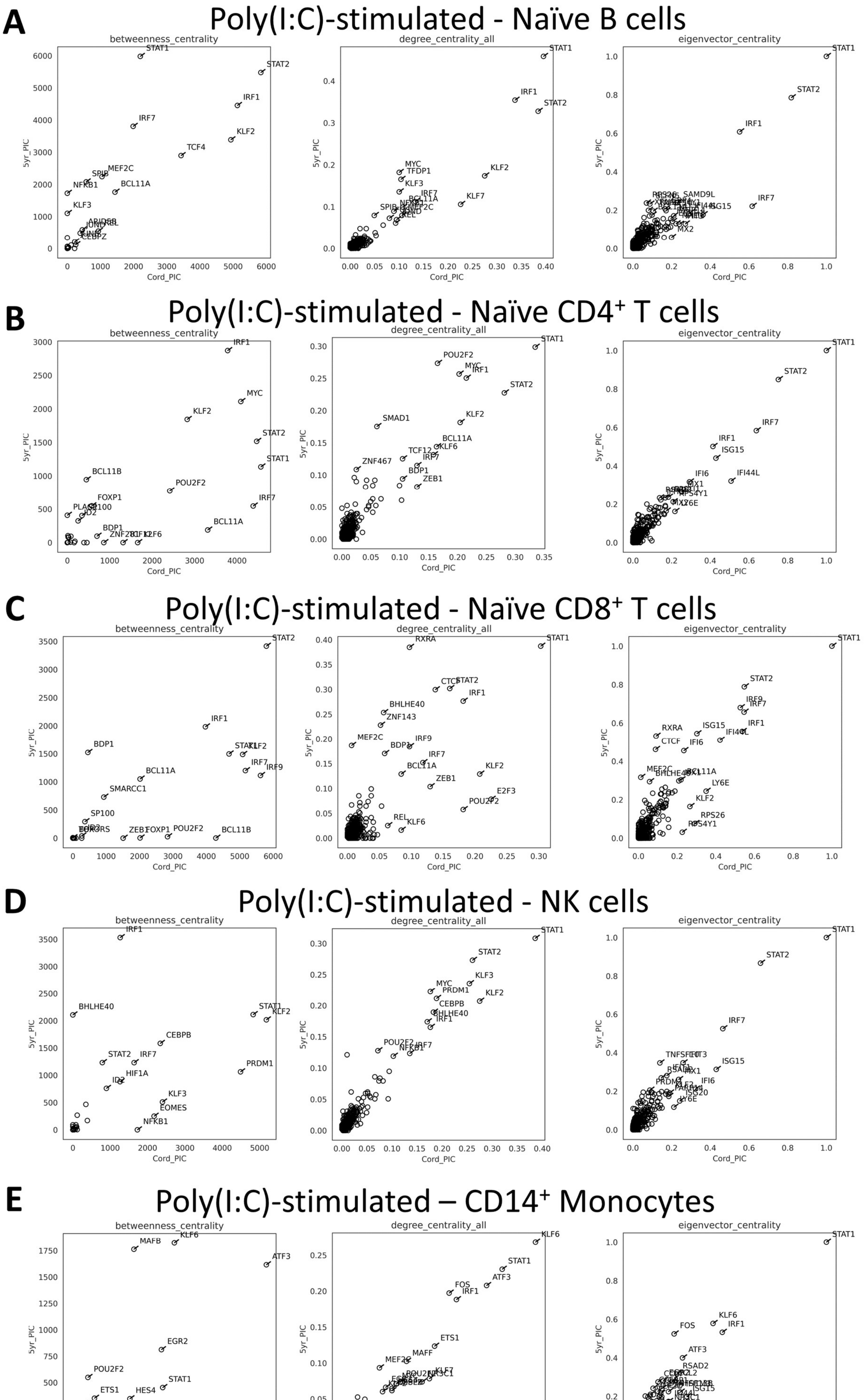

A
Poly(I:C)-stimulated - Naïve B cells
betweenness_centrality
degree_centrality_all
eigenvector_centrality
5yr_PIC
Cord_PIC
B
Poly(I:C)-stimulated - Naïve CD4+ T cells
betweenness_centrality
degree_centrality_all
eigenvector_centrality
5yr_PIC
Cord_PIC
C
Poly(I:C)-stimulated - Naïve CD8+ T cells
betweenness_centrality
degree_centrality_all
eigenvector_centrality
5yr_PIC
Cord_PIC
D
Poly(I:C)-stimulated - NK cells
betweenness_centrality
degree_centrality_all
eigenvector_centrality
5yr_PIC
Cord_PIC
E
Poly(I:C)-stimulated – CD14+ Monocytes
betweenness_centrality
degree_centrality_all
eigenvector_centrality
5yr_PIC
Cord_PIC

Figure S8. Gene Regulatory Network characteristics for selected Poly(I:C)-induced cell types. Plots show the correlation between the metrics for transcription factors and target genes for samples collected from cord blood (CBMC) (x-axis) and 5yr blood (PBMC) (y-axis). Plots are included for naïve B cells (**A**), naïve $CD4^+$ T cells (**B**), naïve $CD8^+$ T cells (**C**), NK cells (**D**), and $CD14^+$ monocytes (**E**), and show the betweenness centrality (left), degree centrality (middle), and eigenvector centrality (right). Plot and metric characteristics are the same as above (Figure S6).

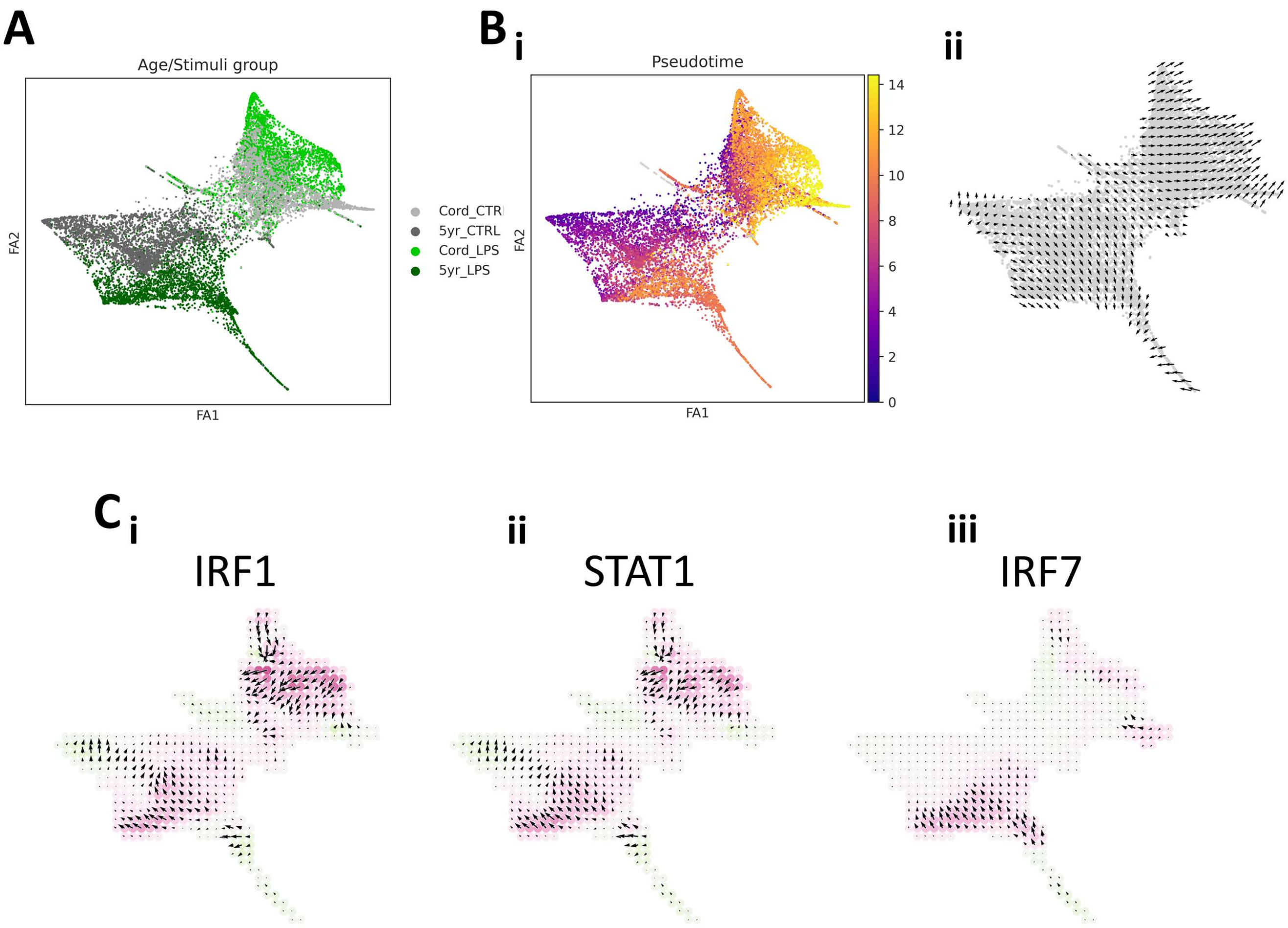

Figure S9. *In silico* perturbation to nullify transcription factor activity (CellOracle). **A**) Force-directed graph of naïve CD4$^+$ T cells stratified by stimuli/age group. This plot recapitulates the characteristics observed from the UMAP (Figure 3B(i)). **B**) **i**) Monocle-defined pseudotime showing differentiation into CBMC-related and 5yr PBMC-related activation states and **ii**) differentiation/activation vectors projected onto the force-directed graph (PAGA). **C**) Knock-out (KO) simulation vector field with perturbation scores for *in silico* KO of *IRF1* (left), *STAT1* (center), and *IRF7* (right). KO of *STAT1* and *IRF1* results in remarkably similar effect on cellular identity. Colors correspond to the perturbation score and denote whether the KO would putatively block (red) or promote (green) activation on that region of the vector field. Arrows indicate the directional change in activation following simulated KO and are calculated as the inner product of the activation trajectory (pseudotime) and the simulated perturbation score. The size of the arrow represents the magnitude of the inner product.

Extended Methods

Materials and Methods

Study subjects

The study was designed to assess matched birth (CBMC) and 5 years (PBMC) blood samples following LPS and Poly(I:C) treatment, along with matched untreated controls, from two donors (one male, one female). The samples were curated from the Childhood Asthma Study (CAS) cohort, a prospective birth cohort for high risk of asthma development(1–5). Cord blood samples were collected from healthy, full-term, singleton births. Matched 5-year samples were collected from the same donor by home visit close to their 5th birthday. This study has ethics approval by The University of Western Australia (reference RA/4/1/7560), and fully informed parental consent was obtained for sample collected from each subject.

In vitro cell culture and innate immune stimulation

Cryopreserved CBMC/5yr PBMC samples were thawed in RPMI 1640 (Gibco) + 10µl DNase (1st freeze/thaw cycle), washed (centrifuged at 400g for 7 mins at RT) and resuspended in 1ml PBS + 2% AB serum (heat inactivated). Residual red blood cells were depleted from cord blood samples with an EasySep RBC Depletion kit (Stemcell Technologies), as per the manufacturer's protocol. Cell viabilities are recorded in Table S1. Cells were resuspended at 1x106 cells per ml in RPMI + 5% AB serum and 0.25x106 cells were transferred to dedicated wells of round bottom 96-well polystyrene culture plates (Thermo Fisher Scientific). Four wells (~1x106 cells) were allocated to each condition. Wells were stimulated with 1ng/ml LPS (Enzo Biochem, Cat No. ALX-581-007-L001, derived from E. coli, serotype R515) or 50µg/ml Poly(I:C) (InvivoGen, Cat. Code: tlrl-pic) or left untreated, and plates were incubated at 37oC (5% CO2) for 18 hours. LPS is a bacterial cell wall component and the quintessential TLR4 ligand. Poly(I:C) is a synthetic analogue of double-stranded RNA (dsRNA) and a potent activator of TLR3 and other nucleic acid sensing receptors (e.g., RIG-I, MDA-5)(6). Each cryopreserved vial was processed on a different day, so that matched stimuli/control samples were processed together. Following culture, replicate culture plate wells (4 per sample/condition) were gently resuspended and transferred to a sterile 1.5ml LoBind tube (Eppendorf). Samples were pelleted (centrifugation at 500 x g for 7 minutes at 4oC) and re-suspended in PBS + 0.04% BSA (UltraPure; Thermo Fisher Scientific) (4oC) to a target concentration of 2,000 cells/µl. Post-culture viability is recorded in Table S1. Samples were immediately transferred on ice to Genomics WA (Perth, Western Australia) for library preparation and sequencing.

Library preparation and sequencing

Single cells were processed on Chromium using the Chromium Next GEM Single Cell 3' Kit v3.1 (4 reactions, PN-1000269, 10X Genomics) on Chip G (PN-1000127, 10x Genomics) according to the manufacturer's protocol with targeted recovery of 5,000 cells per channel. Libraries were sequenced on the NovaSeq 6000 platform in a single batch.

Alignment and initial quality control

Raw fastq.qz files were processed with the CellRanger Toolkit (Version 6.1.1, 10x Genomics) with the Human GRCh38 genome assembly (refdata-gex-GRCh38-2020-A) was used as the reference genome. The CellRanger count pipeline was run with default parameters.

CellRanger web_summary outputs were assessed, and no alerts (warnings or errors) were recorded for any sample. Selected CellRanger outputs are recorded in Table S1; briefly, this project generated (on average) 5,527.17 cells per sample with 63,098.75 mean reads per cell and an average of 1980.17 genes detected per cell, as estimated by CellRanger. The raw feature matrix, and corresponding barcodes and features, were used for downstream QC and analysis.

Sample pre-processing and quality control

Count matrices and corresponding barcode and feature files were imported into the R statistical environment (version 3.6.2), and all subsequent QC/analyses were conducted in R, unless otherwise stated. In general, each sample was run through a QC pipeline which combines functions from several R packages, of which Version 3.2.0 of Seurat(7) was used most extensively for QC, detailed below. The barcodeRanks function from the DropUtils package to compute barcode rank statistics and visualize the knee and inflection points, and the emptyDrops function (DropUtils) was used to identify and retain droplets which putatively contain cells. Droplets were considered cell-containing if their associated barcodes significantly deviate from the ambient profile from 10,000 Monte Carlo iterations (False Discovery Rate < 0.01).

For an initial filter, genes were retained if they were expressed in at least 1% in cells (additional gene filters were implemented for individual methods, below). Next, the quality to individual cells were evaluated and removed based on several metric. Mitochondrial genes were identified by gene symbols with a prefix of "MT-" and ribosomal genes were identified with a "RPS" or "RPL" prefix. Quality control metrics were calculated for each cell with the addPerCellQC function from the scuttle package. Cells were excluded if they had less than 500 unique gene detected and/or less that 1000 total transcripts. Cells were also excluded if they had a mitochondrial gene content greater than three median absolute deviations (MADs) above the median. This adaptive threshold was chosen to capture the mitochondrial content distribution of each sample independently, rather than applying a constant threshold (e.g., 5-10%) across all samples. Additionally, a threshold of 50% ribosomal content was used to exclude cells with a majority of their gene expression accounted for by ribosomal genes (which excluded few cells).

Next, The standard Seurat pipeline was applied to explore and asses each sample. Gene expression was log normalized with the NormalizeData function (scale.factor = 10000), and the top 2,000 most variable features were identified with the FindVariableFeatures function (selection.method = "vst"). The data was scaled and centered with the ScaleData function, and used for an initial principal component dimensionality reduction was applied with the RunPCA function (npcs = 50) using the top 2,000 variable genes. The VizDimLoadings and DimPlot functions were used to assess the loading contribution of genes and plot the first two PCs, respectively. Additionally, the DimHeatmap function (cells = 500, balanced = TRUE) was used to visualize the first 9 PCs.

To assess whether the each sample displayed discrete clusters from their gene expression profiles, the FindNeighbors (reduction = "pca", dims = 1:15, k.param = 20, n.trees = 50, annoy.metric = "euclidean") and FindClusters (resolution = 0.2 n.start = 10, n.iter = 10) functions were used to find the 20 nearest neighbors and apply a nearest neighbor modularity

optimization clustering algorithm to identify distinct clusters, respectively. Next, the Uniform Manifold Approximation and Projection (UMAP) approach was applied to reduce the multi-dimensional data (previously calculated PCs) to 2-Dimensional coordinates for the purpose of visualization using the RunUMAP function (reduction = "pca", umap.method = "uwot", n.neighbors = 30, n.components = 2, metric = "cosine", learning.rate = 1, min.dist = 0.3, spread = 1, local.connectivity = 1, repulsion.strength = 1, negative.sample.rate = 5, dens.lambda = 2, dens.frac = 0.3, dens.var.shift = 0.1). The resulting UMAP representation of the data were plotted with the DimPlot function with individual cell colored according to which cluster they were assigned. Additionally, the DoHeatmap function was used to plot the 10 genes per cluster. From the above cluster analysis, each sample exhibited discrete clustering as expected from gene expression profiles of mix cell populations.

The CellCycleScoring function was used to estimate the cell cycle phase of each cell for each sample using the reference cell cycle marker genes loaded with the Seurat package. Doublets were detected and removed with DoubletFinder(8) package. First, the paramSweep_v3 (PCs = 1:15, sct = FALSE) and summarizeSweep (GT = FALSE) functions to perform an artificial doublets (pN) and neighborhood sizes (pK) parameter sweep, summarized the outputs, and compute the bimodality coefficient for each pK values. The optimum pK values was assigned to the value with the highest corresponding bimodality coefficient. The doubletFinder_v3 function (PCs = 1:15, pN = 0.25, pK = optimum_pK_value, reuse.pANN = FALSE, sct = FALSE) was run with an assumption of a 5% doublet rate from all cells, and cells assigned as a doublet were excluded.

To assess whether expected cell types were present in each of the CBMC/PBMC samples, gene expression of selected marker genes (*CD3E*, *CD4*, *CD8A*, *NKG7*, *GNLY*, *CD14*, *LYZ*, *CD19*, *CD79A*, and *CD34*) was plotted with the DimPlot function. Additionally, unbiased reference-based annotation of each cell was applied with singleR(9). The high-level annotation were used from both the Human Primary Cell Atlas and Blueprint Encode references provided. From this assessment, each sample displayed distinct clusters which could be attributed to major cell types expected in the circulating blood ($CD4^+$ T cells, $CD8^+$ T cells, B cells, NK cells and Monocytes). All sample were assesses as good quality and the pre-processing and quality control metrics are recorded in **Table S1**. Raw counts data (post CellRanger) are available via the Gene Expression Omnibus (GSE232186).

Integration, Annotation, and Dimensionality reduction

The integration method from the open-source R toolkit Seurat(7) was used to integrate individual samples following quality control. Pre-processed samples were converted to a list object and the raw counts from each sample was log normalised with the NormalizeData function (scale.factor = 10000, margin = 1). The top 2,000 variable features were re-calculated with the FindVariableFeatures (selection.method = "vst") and the ScaleData function was used to scale and center the log normalised data. The first 50 principal components were calculated for each samples with the RunPCA function using the top 2,000 variable features.

The FindIntegrationAnchors function (anchor.features = 2000, scale = TRUE, normalization.method = "LogNormalize", reduction = "rpca", l2.norm = TRUE, dims = 1:30, k.anchor = 5, k.filter = 200, k.score = 30, max.features = 200, nn.method = "annoy", n.trees = 50, eps = 0) was used to identify 2,000 'anchors'. Anchors are cell-cell pairs with a

correspondent similarity between two data sets that are expected to exhibit a common set of molecular features (i.e., cells with near identical gene expression profiles in different data sets). The process of identifying anchors is described in depth in the original publication(7); Briefly, data set dimensionality is reduced with diagonalized Canonical Correlation Analysis and L2 normalization (scaling so that the sum of the squares sums to 1) is applied to the output vectors. Anchors are then identified as mutual nearest-neighbors in the reduced dimensional representation. These anchors were used to integrate the data with the IntegrateData function (n ormalization.method = "LogNormalize", dims = 1:30, k.weight = 100, weight.reduction = NULL, sd.weight = 1, eps = 0). For this integration, anchors are scored between reference and query data sets, and the difference in expression profiles of the anchor cells are used to apply a weighted average correction transformation(7).

Individual cells were annotated with Azimuth(10), a web-based application which provides reference-based mapping to unbiasedly annotate scRNA-Seq profiles. The annotation approach employed vai Azimuth is detailed in the original publication(10) (in particular, see 'Reference-based Integration for query datasets' in the Methods section). Briefly, the counts data (query data set) was transformed to the same low-dimensional space (supervised PCA) as a reference data set, and the cell type labels from the reference data set are transferred to corresponding cells of the query data set, along with a prediction/confidence score. For this study, the human PBMC reference data set was used as the reference; a CITE-seq dataset with gene expression for hundreds of thousands of cells alongside a large panel of antibodies which was used to accurately phenotype cell types present in the PBMC from cell surface markers. The level 2 cell type resolution was used, and cells were excluded if they had a prediction/confidence score less than 0.5. Following integration and annotation, the pheatmap function was used to create a heatmap to display the number of cells detected for each cell types, by various stratifications (e.g., Age/stimuli, Donor, etc).

Uniform Manifold Approximation and Projection (UMAP) was employed for dimensionality reduction. First, the ScaleData function was used to scale and center the integrated data set, and the first 30 principal components were calculated with the RunPCA function (). Although non-linear dimensionality reduction methods such as UMAP are widely used to visualize relationships between cells within high dimensional data in 2-Dimensional space (e.g., cell type clusters in scRNA-Seq data sets), these methods often do so at the cost of preservation of the local and/or global structure of the data(11). We wanted to tested whether the plots generated from our UMAP analysis were robust to different values of two of the most influential parameters for UMAP embedding; the number of nearest neighbors (n.neighbors) and the minimum distance (min.dist) between cells. To do this, the RunUMAP function was used to generate 2-dimensional representations of the data with combinations of nearest neighbour values of 5, 10, 15, 20, 30, 40, 50 and minimum distance values of 0.1, 0.2, 0.3, 0.4, and 0.5, and other parameter set as follows; map.method = "uwot", n.components = 2, metric = "cosine", n.epochs = NULL, learning.rate = 1, spread = 1, set.op.mix.ratio = 1, local.connectivity = 1, repulsion.strength = 1, negative.sample.rate = 5. The nearest neighbour and minimum distance values were chosen as they span the suggested range for these parameters. The DimPlot function was used to plot the first two UMAP components of each UMAP construction and the plot with nearest neighbour value of 20 and a minimum distance values of 0.3 was selected as a representative for visualisation purposes to display the

integrated cell type clustering overlayered with Azimuth-defined annotations, as well as relevant experimental group variables and marker gene expression intensities.

Differential gene expression and Pathways analysis

To identify differentially expressed genes (DEGs) between LPS/Poly(I:C) and corresponding unstimulated control samples for each cell type, we employed Model-based Analysis of Single-cell Transcriptomics (MAST)(12) via the FindMarkers function from Seurat. MAST employs a two-part (hurdle) generalized linear model tailored to address the bimodal, zero-inflated gene expression distributions encountered in scRNA-seq data. The cellular detection rate (CDR, the fraction of genes expressed in each cell), mitochondrial gene proportion, and cell cycle phase were included in as covariates for MAST analysis. As each donor represents a different biological sex (male, female), this variable was also included as a latent variable. This approach was selected to accommodate our small sample size (two biological donors), although we acknowledge that incorporating individual participant variation as a latent variable is a suboptimal approach compared to other methods suited to larger sample sizes (viz. pseudobulk and mixed models with a random effect for individual)(13,14), and this is a limitation of our study. However, our analysis identified many co-regulated genes which would be expected to be dysregulated following treatment with LPS and Poly(I:C) (corroborated by pathways enrichment analysis and independently identified with GRN analysis) and the primary genes of interest recorded extremely small Bonferroni-corrected p values (commonly $< 10x10^{-20}$). For these reasons, we believe the loss in precision of the MAST + latent variable method had limited impact on the findings in our study.

Genes were considered differentially expressed if they recorded a Bonferroni-corrected p value < 0.01 and an average Log2 fold-change in expression of > 0.25 (upregulated) or < -0.25 (downregulated). Aligned volcano plots displaying DEGs identified from multiple cell type simultaneously were plotted in base R and DEG count heatmaps were plotted with the pheatmap function in R. The overlap of differentially expressed genes were plotted with the upset functions from the UpSetR package.

Functional biological pathways associated with significant DEGs between cell type/stimuli groups were identified with the enrichment analysis tool enrichR(15–17) in R. EnrichR calculates enrichment of an input gene set within the annotate gene sets of functional pathways (curated from >200 databases) with Fisher exact test (and variations thereof(15)), and provides many additional analysis and plotting tools. Significant DEG sets (up- and down-regulated genes separately) were used to query biologically-relevant annotated gene sets for significant enrichment from the Reactome(18), KEGG(19), and Gene Ontology(20) databases. These databases were selected as they are well established and offer a wide range of biological functions, including those related to the innate immune response. Significantly enriched pathways from all databases were ordered by decreasing $-Log_{10}$ p values and plotted as a bar plot with base R.

Pseudotime trajectory inference

We applied Monocle3(21) to infer stimuli-related activation trajectories from transitional cellular states present in the data. For each analysis, only the raw counts from cells relevant for that comparison (e.g., CBMC/5yr PBMC untreated and LPS-treated monocytes) were included. The count data was pre-processed with the preprocess_cds function (method =

"PCA", num_dim = 50, norm_method = "log") and the cells were aligned with the align_cds function (preprocess_method = "PCA", alignment_k = 20) with the donor variable used as the alignment group and the cellular detection rate and mitochondrial content included as terms in the model formula. Monocle3 require the multidimensional gene expression data to be projected to a lower dimensional representation to learn and fit putative trajectories of an underlying biological process. For this purpose, UMAP dimensionality reductions were generated with the reduce_dimension function with 10 nearest neighbours and a minimum distance of 0.1, and cells were clustered with a k value (nearest neighbours) of 10 and a resolution value of 0.001 for all comparisons, with the exception of cluster resolution values of 0.002 and 0.005 for LPS- and Poly(I:C)-induced naïve CD4+ T cells comparisons, respectively, to accommodate larger cell numbers. Other parameters for this function were max_components = 2, reduction_method = "UMAP", umap.metric = "cosine", umap.fast_sgd = FALSE, umap.nn_method = "annoy". Unsupervised clustering was performed with the cluster_cells function (reduction_method = "UMAP", k = 10, cluster_method = "leiden", num_iter = 2, partition_qval = 0.01, weight = FALSE, resolution = 0.001), which uses the Leiden (or Louvain, if selected) community detection method. A principle graph was fitted to data with the learn_graph function (use_partition = TRUE, close_loop = FALSE) and cells were assigned a pseudotime value based on their projection on the principal graph with the order_cells function (reduction_method = "UMAP", root_pr_nodes = NULL, root_cells = NULL). Regions enriched with unstimulated controls were selected as pseudotime start points so that trajectories extended into stimuli-activated regions. The reduced dimension representation displaying experimental groups and the assigned pseudotime values were plotted with the plot_cells function.

Gene Regulatory Network (GRN) analysis and in silico perturbations

We employed CellOracle(22) to build GRNs in order to identify the key molecular drivers (transcription factors (TF)) and their corresponding target genes for selected cell type/stimuli groupings. For this analysis, SCANPY(23) (version 1.9.3) was used for pre-processing and force directed graph construction (Partition-based graph abstraction (PAGA)(24)) and CellOracle (version 0.12.0) was run with Python (3.10.6) on Ubuntu 22.04.1 via Windows Subsystem for Linux 2 kernel. Genes with at least 1 count were retained and then genes were filters to identify the top 3000 most variable for each comparison. The data was normalized (scanpy.pp.normalize_per_cell), log transformed (scanpy.pp.log1p) and scaled (scanpy.pp.scale) with default parameters. Additionally, the donor variable was adjusted for in the data with scanpy.pp.regress_out. Separate analyses were run from raw counts for each cell type/stimuli comparison and group specific GRNs (e.g., Cord_Control, Cord_LPS, 5yr_Control, and 5yr_LPS) were constructed from the Human promoter base GRN provided. CellOracle was run with standard parameters and the monocle3-defined pseudotime values for each cell were included for analysis. From the output of CellOracle, we plotted Venn diagrams (ggvenn R package) to show the overlap of significantly connected (p value < 0.01) target genes (TG) of IRF1, IRF7, STAT1, and STAT2 for each comparison, and displayed TF-TG wiring diagrams of the top 100 TG connections with the igraph R package. CellOracle constructed GRNs were then used to perform in silico transcription factor perturbations of IRF1, IRF7, and STAT1 to simulate the changes in cellular states after nullifying the regulatory

effects of the TFs. The scale parameter was adjusted to suit each comparison (as recommended) and standard parameters were used for all other functions in this analysis.

Ligand-Receptor interaction analysis

We employed CellCall(25) to identify putative ligand-receptor (L-R) communication between selected cell types following LPS- and Poly(I:C)-induced activation. For each stimuli/age comparison, the data set was subset to selected cell types of interest (B naïve, CD14 Mono, CD16 Mono, CD4 TCM, CD4 Naïve, CD8 Naïve, CD8 TEM, HSPC, ILC, NK, Treg), and genes were filtered to the top 2000 most variable (compared to corresponding unstimulated control samples) with the FindVariableFeatures function from Seurat (selection.method = "vst", loess.span = 0.3). Raw counts were incorporated into a xxx with the CreateNichConObject function (min.feature = 3, names.field = 1, scale.factor = 10^6), and the transcriptional communication profile was calculated with the TransCommuProfile function (pValueCor = 0.05, CorValue = 0.1, topTargetCor=1, p.adjust = 0.1, use.type="mean", probs = 0.1, method="weighted"). L-R interactions stratified by cell type were visualized as circus plots with the ViewInterCircos function. The LR2TF function was used to extend the analysis by assigning putatively activated TFs downstream of receiver cell receptor binding of sender cell ligands for communication between $CD14^+$ monocytes and naïve $CD4^+$ T cells. The Ligand-Receptor-TF relationships were visualized as Sankey plots with the sankey_graph function.

Supplementary references:

1. Heaton T, Rowe J, Turner S, Aalberse RC, de Klerk N, Suriyaarachchi D, et al. An immunoepidemiological approach to asthma: identification of in-vitro T-cell response patterns associated with different wheezing phenotypes in children. Lancet. 2005 Jan 8;365(9454):142–9.
2. Kusel MMH, de Klerk NH, Holt PG, Kebadze T, Johnston SL, Sly PD. Role of respiratory viruses in acute upper and lower respiratory tract illness in the first year of life: a birth cohort study. Pediatr Infect Dis J. 2006 Aug;25(8):680–6.
3. Kusel MMH, de Klerk NH, Kebadze T, Vohma V, Holt PG, Johnston SL, et al. Early-life respiratory viral infections, atopic sensitization, and risk of subsequent development of persistent asthma. J Allergy Clin Immunol. 2007 May;119(5):1105–10.
4. Holt PG, Rowe J, Kusel M, Parsons F, Hollams EM, Bosco A, et al. Toward improved prediction of risk for atopy and asthma among preschoolers: a prospective cohort study. J Allergy Clin Immunol. 2010 Mar;125(3):653–9, 659.e1-659.e7.
5. Holt PG, Mok D, Panda D, Renn L, Fabozzi G, deKlerk NH, et al. Developmental regulation of type 1 and type 3 interferon production and risk for infant infections and asthma development. J Allergy Clin Immunol. 2019 Mar;143(3):1176-1182.e5.
6. Yu M, Levine SJ. Toll-like receptor, RIG-I-like receptors and the NLRP3 inflammasome: key modulators of innate immune responses to double-stranded RNA viruses. Cytokine Growth Factor Rev. 2011 Apr;22(2):63–72.
7. Stuart T, Butler A, Hoffman P, Hafemeister C, Papalexi E, Mauck WM, et al. Comprehensive Integration of Single-Cell Data. Cell. 2019 Jun 13;177(7):1888-1902.e21.

8. McGinnis CS, Murrow LM, Gartner ZJ. DoubletFinder: Doublet Detection in Single-Cell RNA Sequencing Data Using Artificial Nearest Neighbors. Cell Syst. 2019 Apr 24;8(4):329-337.e4.
9. Aran D, Looney AP, Liu L, Wu E, Fong V, Hsu A, et al. Reference-based analysis of lung single-cell sequencing reveals a transitional profibrotic macrophage. Nat Immunol. 2019 Feb;20(2):163–72.
10. Hao Y, Hao S, Andersen-Nissen E, Mauck WM, Zheng S, Butler A, et al. Integrated analysis of multimodal single-cell data. Cell. 2021 Jun 24;184(13):3573-3587.e29.
11. Chari T, Pachter L. The specious art of single-cell genomics. PLoS Comput Biol. 2023 Aug;19(8):e1011288.
12. Finak G, McDavid A, Yajima M, Deng J, Gersuk V, Shalek AK, et al. MAST: a flexible statistical framework for assessing transcriptional changes and characterizing heterogeneity in single-cell RNA sequencing data. Genome Biol. 2015 Dec 10;16:278.
13. Zimmerman KD, Espeland MA, Langefeld CD. A practical solution to pseudoreplication bias in single-cell studies. Nat Commun. 2021 Feb 2;12(1):738.
14. Junttila S, Smolander J, Elo LL. Benchmarking methods for detecting differential states between conditions from multi-subject single-cell RNA-seq data. Brief Bioinform. 2022 Sep 20;23(5):bbac286.
15. Chen EY, Tan CM, Kou Y, Duan Q, Wang Z, Meirelles GV, et al. Enrichr: interactive and collaborative HTML5 gene list enrichment analysis tool. BMC Bioinformatics. 2013 Apr 15;14:128.
16. Kuleshov MV, Jones MR, Rouillard AD, Fernandez NF, Duan Q, Wang Z, et al. Enrichr: a comprehensive gene set enrichment analysis web server 2016 update. Nucleic Acids Res. 2016 Jul 8;44(W1):W90-97.
17. Xie Z, Bailey A, Kuleshov MV, Clarke DJB, Evangelista JE, Jenkins SL, et al. Gene Set Knowledge Discovery with Enrichr. Curr Protoc. 2021 Mar;1(3):e90.
18. Fabregat A, Jupe S, Matthews L, Sidiropoulos K, Gillespie M, Garapati P, et al. The Reactome Pathway Knowledgebase. Nucleic Acids Res. 2018 Jan 4;46(D1):D649–55.
19. Kanehisa M, Goto S. KEGG: kyoto encyclopedia of genes and genomes. Nucleic Acids Res. 2000 Jan 1;28(1):27–30.
20. Ashburner M, Ball CA, Blake JA, Botstein D, Butler H, Cherry JM, et al. Gene ontology: tool for the unification of biology. The Gene Ontology Consortium. Nat Genet. 2000 May;25(1):25–9.
21. Cao J, Spielmann M, Qiu X, Huang X, Ibrahim DM, Hill AJ, et al. The single-cell transcriptional landscape of mammalian organogenesis. Nature. 2019 Feb;566(7745):496–502.
22. Kamimoto K, Stringa B, Hoffmann CM, Jindal K, Solnica-Krezel L, Morris SA. Dissecting cell identity via network inference and in silico gene perturbation. Nature. 2023 Feb;614(7949):742–51.
23. Wolf FA, Angerer P, Theis FJ. SCANPY: large-scale single-cell gene expression data analysis. Genome Biol. 2018 Feb 6;19(1):15.
24. Wolf FA, Hamey FK, Plass M, Solana J, Dahlin JS, Göttgens B, et al. PAGA: graph abstraction reconciles clustering with trajectory inference through a topology preserving map of single cells. Genome Biol. 2019 Mar 19;20(1):59.

25. Zhang Y, Liu T, Hu X, Wang M, Wang J, Zou B, et al. CellCall: integrating paired ligand-receptor and transcription factor activities for cell-cell communication. Nucleic Acids Res. 2021 Sep 7;49(15):8520–34.
